# Engineering Dirac Cones in Graphene for Efficient Iodide Catalysis: Insights by Nanoscale Electrochemistry

**DOI:** 10.1002/advs.76835

**Published:** 2026-08-10

**Authors:** Yang‐Sheng Lu, Kuo‐En Chang, Ta‐Lei Chou, Aitor Garcia‐Ruiz, Ting‐Han Lin, Yen‐Ting Liu, Akichika Kumatani, Ming‐Hao Liu, Kenji Watanabe, Takashi Taniguchi, Ming‐Wen Chu, Tse‐Ming Chen, Chun‐Wei Chen, Shao‐Sian Li

**Affiliations:** ^1^ Department of Materials and Mineral Resources Engineering National Taipei University of Technology Taipei Taiwan; ^2^ Center of Atomic Initiative for New Materials National Taiwan University Taipei Taiwan; ^3^ Department of Physics National Cheng Kung University Tainan Taiwan; ^4^ Center for Quantum Frontiers of Research & Technology (QFort) National Cheng Kung University Tainan Taiwan; ^5^ Center for Condensed Matter Sciences National Taiwan University Taipei Taiwan; ^6^ Department of Electrical and Electronic Engineering Chiba Institute of Technology Chiba Japan; ^7^ WPI‐Advanced Institute of Materials Research and Graduate School of Engineering Tohoku University Sendai Japan; ^8^ Research Center for Electronic and Optical Materials National Institute for Materials Science Tsukuba Ibaraki Japan; ^9^ Research Center for Materials Nanoarchitectonics National Institute for Materials Science Tsukuba Ibaraki Japan; ^10^ Department of Materials Science and Engineering National Taiwan University Taipei Taiwan

**Keywords:** dirac cone engineering, graphene gating, iodine oxidation, magic‐angle twisted bilayer graphene, nanoscale electrochemistry, sustainable electrocatalysts

## Abstract

Advances in atomically thin two‐dimensional materials have created new opportunities to regulate interfacial charge transfer and surface reactivity in electrocatalysis. Among anodic reactions relevant to sustainable energy conversion, the iodide oxidation reaction (IOR) has emerged as an attractive alternative to the oxygen evolution reaction because of its lower overpotential, faster kinetics, and simultaneous generation of value‐added iodine species. Here, by using scanning electrochemical cell microscopy, we show that the electronic structure of graphene can be engineered to promote IOR through two experimentally distinct routes: electrostatic gating in monolayer graphene and twist‐angle control in bilayer graphene. Gate‐dependent measurements show that hole doping promotes iodide oxidation, whereas electron doping suppresses it, consistent with Fermi‐level modulation of electron extraction from iodide. Magic‐angle twisted bilayer graphene (MATBG) exhibits a lower onset potential and stronger IOR response than monolayer and Bernal‐stacked bilayer graphene. The enhanced activity of MATBG is associated with a moiré‐reconstructed interfacial electronic environment that facilitates IOR. These results establish electronic‐structure engineering of graphene as an effective strategy for regulating nanoscale iodide oxidation and highlight Dirac‐cone engineering as a tunable platform for sustainable iodine redox electrochemistry.

## Graphical abstract

Graphene iodide oxidation is regulated by Dirac cone engineering. Electrostatic gating tunes the Fermi level to control local iodide oxidation reaction activity, while magic‐angle twisted bilayer graphene creates flat‐band‐derived interfacial states that promote charge transfer and iodine‐species interaction, revealing a nanoscale route to electronic‐structure‐driven catalysis.

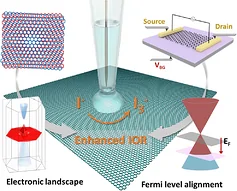

## 1 Introduction

Conventional catalyst design has primarily focused on strategies such as elemental doping [1, 2], defect engineering [3, 4, 5], and heterostructure construction [6, 7, 8] to modulate surface reactivity. Beyond these approaches, tuning of the electronic structure has emerged as a highly effective strategy, as it directly influences charge redistribution, intermediate adsorption energetics, and catalytic reaction kinetics [9, 10, 11]. In particular, for two‐dimensional (2D) materials, their atomically thin nature and highly tunable band structures make electronic structure engineering for rational catalyst optimization particularly effective, offering a new opportunity to regulate interfacial charge transfer and surface reactivity in catalytic reactions [12, 13, 14, 15].

The oxygen evolution reaction (OER) has long been recognized as the anodic half‐reaction in water splitting for hydrogen production and related electrochemical energy conversion systems [16, 17]. However, the OER is intrinsically sluggish, as it requires a complex four‐electron transfer process coupled with O─O bond formation [18]. These multi‐step pathways lead to substantial overpotentials, slow kinetics, and significant energy losses, rendering OER the performance‐limiting step in most electrochemical and photoelectrochemical devices [19, 20, 21]. By contrast, the iodide oxidation reaction (IOR) offers a kinetically and energetically more favorable alternative [17, 22, 23, 24]. The oxidation of iodide to triiodide(I_3_^−^) or iodine typically proceeds through a two‐electron pathway with much faster kinetics and considerably lower overpotential [22, 24]. The theoretical standard redox potential of the I^−^/I_3_^−^ couple (+0.54 V vs. SHE, Standard Hydrogen Electrode) is significantly lower than that of OER (+1.23 V vs. SHE) [25], enabling IOR to operate at reduced cell voltages [17]. Recent progress in iodine redox chemistry has further highlighted the relevance of iodide/iodine conversion beyond electrolysis, including iodine‐based energy‐storage systems, redox‐mediated energy devices, and IOR‐coupled hydrogen production [26, 27, 28, 29, 30, 31]. In addition, iodine (I_2_) is an important compound due to its wide range of applications, including use as a catalyst in chemical synthesis, in pharmaceuticals, food supplements, photography, spacecraft propulsion, and more [32, 33, 34, 35]. Consequently, the low onset potential of the IOR makes it an attractive alternative for the anodic half‐reaction in water splitting, as it not only reduces the energy required for hydrogen production but also enables the formation of high‐value‐added products, rather than O_2_ [36, 37, 38, 39].

Compared to conventional IOR catalysts such as transition metals [23, 36, 38] or noble metals [40], a recent discovery has shown that carbon‐based materials can act as efficient, low‐cost metal‐free catalysts for the IOR, featuring a low oxidation potential [22]. The impressive catalytic performance is attributed to the delocalized π‐electron system in graphite‐based materials, which facilitates rapid charge transfer with iodine species [22]. Graphene, a 2D material composed of a single layer of sp^2^‐hybridized carbon atoms, exhibits exceptional electronic properties arising from its linear energy dispersion and high carrier mobility, which are described by Dirac cones [41, 42]. The electronic structure of graphene can be modulated by manipulating the Dirac‐cone position and shape through electrostatic gate control [43, 44, 45], twist angle in bilayer graphene [46, 47], or structural modification by strain [48, 49] to tailor carrier density, band alignment, and quantum states near the Fermi level. Among these strategies, electrostatic gating shifts the Fermi level relative to the Dirac point and dynamically tunes the carrier density and charge‐transfer barrier [50, 51]. Twisted bilayer graphene (TBG), especially near the so‐called magic angle (∼1.1°), forms moiré superlattices with spatially varying AA/AB/BA stacking environments and flat‐band‐associated electronic localization [46, 47, 52, 53]. These moiré‐engineered states create a spatially heterogeneous interfacial electronic environment in which AA‐enriched regions could significantly enhance electrochemical charge transfer [46, 47, 54] and influence the adsorption/desorption kinetics of iodine‐related intermediate species [22, 55, 56]. In this work, we use scanning electrochemical cell microscopy (SECCM) to compare two experimentally distinct approaches for Dirac‐cone engineering in graphene—electrostatic gating in monolayer graphene and twist‐angle control in bilayer graphene—and to determine how each route modulates local IOR behavior [57, 58]. Electrostatic gating provides a continuously tunable route to modulate the Fermi level and carrier polarity of monolayer graphene. In contrast, magic‐angle twisted bilayer graphene (MATBG) offers structural modulation that generates a moiré‐reconstructed interfacial electronic environment. Examining both platforms within the same SECCM framework allows us to explore the effects of manipulating the electronic structures of graphene—via either continuous Fermi‐level tuning or moiré electronic reconstruction—to facilitate the IOR. In addition, because SECCM probes a confined nanoscale meniscus rather than a macroscopic cell, it is particularly well suited to interrogating collective microscopic electrochemical responses in structurally heterogeneous graphene systems such as TBG [46]. This framework allows us to discuss graphene IOR catalysis beyond a simple density of states (DOS) argument and instead in terms of coupled electronic‐structure tuning, moiré‐scale heterogeneity, and iodine‐species interfacial chemistry.

## 2 Results and Discussion

Figure 1a presents the schematic setup of SECCM for IOR measurements. In this experiment, a scanning nanopipette probe containing 50 mm H_2_SO_4_ + 0.1 m NaI and a palladium (Pd) wire is used as a localized electrochemical cell, where the Pd wire functions as a quasi‐reference counter electrode (QRCE) that both completes the local electrochemical circuit and provides the potential reference for the voltammetric measurement. This electrolyte composition was selected to provide a stable iodide‐containing acidic meniscus and a reproducible faradaic response during localized voltammetry. Upon meniscus contact with the graphene surface, localized linear sweep voltammetry (LSV) is performed, and the faradaic current associated with IOR is recorded. The nanoprobe enables localized discrimination and spatial visualization of electrochemical currents with a lateral resolution of approximately 100 nm. Details of SECCM probe fabrication, measurement conditions, and calibration are provided in the Supporting Information. This technique has previously been employed to differentiate the nanoscale electrochemical origins between the active edge and the basal plane in 2D monolayer MoS_2_ and WS_2_ catalysts [33, 58]. Here, we employed high‐quality monolayer graphene synthesized via chemical vapor deposition (CVD) and subsequently transferred onto n^+^‐Si substrates for SECCM measurements. The quality and uniformity of the graphene were characterized by optical microscopy, Raman spectroscopy, and Atomic Force Microscopy (AFM), as shown in Figures S1–S3. Raman spectroscopy reveals a sharp 2D band with a 2D‐to‐G ratio of ∼2 and negligible D‐band intensity, indicating high‐quality CVD‐grown graphene. The optical image and AFM analysis show a continuous, void‐free morphology, with a thickness (∼0.83 nm) matching pristine monolayer graphene. These results demonstrate that the transfer process onto the n^+^‐Si substrate preserves both the quality and continuity of the film.

**FIGURE 1:**
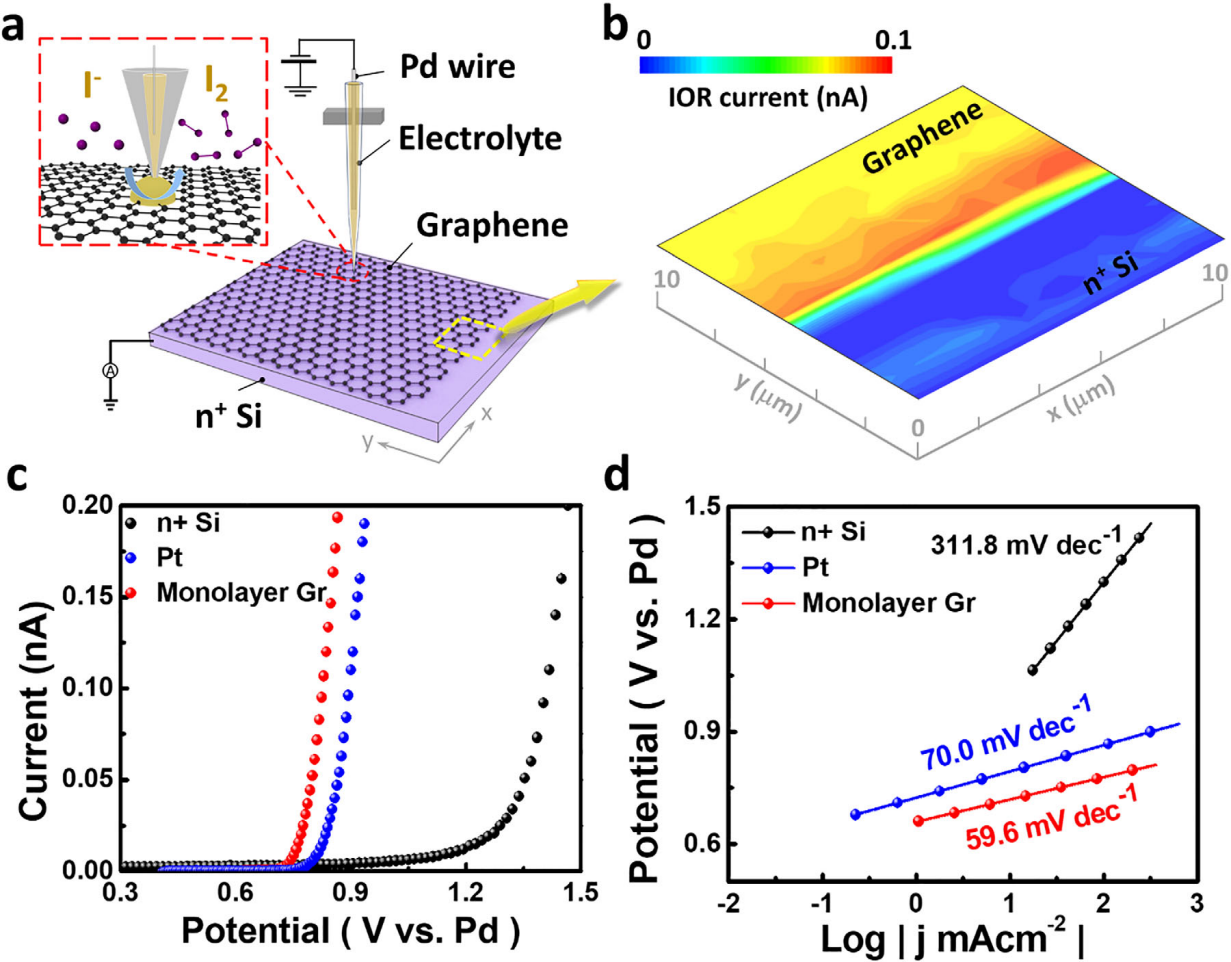
(a) Schematic illustrations of SECCM measurements conducted on monolayer graphene for the IOR. The nanopipette contains 50 mm H_2_SO_4_ + 0.1 m NaI and a Pd wire QRCE, which completes the local electrochemical circuit and provides the potential reference. (b) Spatially resolved IOR current map on the monolayer graphene/SiO_2_ interface as indicated by the yellow square in (a). (c) SECCM‐acquired LSV curves comparing the IOR behavior of n^+^Si, Pt, and monolayer graphene. The corresponding Tafel‐slope analysis is presented in (d).

Figure 1b presents the normalized SECCM current map for monolayer graphene transferred onto an n^+^‐Si substrate containing adjacent graphene‐covered and bare regions as a control. The graphene region exhibits strong IOR currents, whereas the bare n^+^‐Si shows no detectable response, resulting in a sharp contrast at the interface. The current map was acquired at an applied potential of 0.8 V vs. a Pd wire to probe the IOR while avoiding any contribution from the OER, which typically initiates on graphene at ∼1.3–1.4 V vs. Pd (see Supporting Information). Consequently, the contrast observed in Figure 1b arises solely from the IOR. A reduced IOR current was observed near the graphene edge because the collected response includes mixed contributions from both graphene and the inactive Si substrate. Figure 1c shows the corresponding LSV curves for graphene, Pt, and n^+^‐Si, which reflect the efficiency of interfacial electron transfer driving the IOR within the nanoscale electrochemical cell. Owing to the delocalized π‐electron network in its honeycomb carbon lattice, graphene facilitates efficient interfacial charge transfer for IOR, resulting in a lower onset potential of ∼0.75 V vs. Pd than that of Pt (∼0.82 V vs. Pd) under similar conditions. In contrast, the n^+^‐Si substrate shows a much higher onset potential of ∼1.2 V vs. Pd. The local reaction kinetics of IOR were further examined by Tafel analysis, as shown in Figure 1d. The monolayer graphene sample exhibits a Tafel slope of 59.6 mV dec^−^^1^, notably smaller than that of Pt (70.0 mV dec^−^^1^) under similar conditions, consistent with more facile local IOR kinetics on graphene. It is important to note that the SECCM IOR responses are not fully iR‐corrected because the iR‐drop contribution is difficult to precisely determine in the confined nanopipette/meniscus geometry. To minimize uncontrolled variation, all measurements were performed using the same SECCM configuration, and a nanopipette pre‐screening procedure was applied to reduce probe‐to‐probe differences in pipette geometry and effective meniscus response (see Supporting Information). Therefore, the reported values should be interpreted as experimentally measured local responses that allow consistent comparison of relative IOR trends among different graphene samples and experimental conditions. To verify the reproducibility of the SECCM observations throughout this work, all measurements are carried out at multiple locations.

Electrostatic gating has recently been proposed to account for the remarkable catalytic activity of ultrathin semiconductors, such as 2D transition metal dichalcogenides (TMDs) [59, 60, 61, 62]. Although semiconductors are often regarded as nonideal catalysts due to their intrinsically low carrier concentrations, their surface carrier density can be strongly modulated by electrolyte gating during electrocatalytic operation. As a result, n‐type 2D semiconductor catalysts typically favor cathodic reactions, such as the hydrogen evolution reaction (HER), whereas p‐type counterparts are more effective for anodic reactions, such as the oxygen evolution [11, 62]. By contrast, graphene exhibits a distinctive electronic structure characterized by Dirac cones, where the conduction and valence bands intersect linearly at the Dirac point. The Fermi level of graphene can be tuned through electrostatic gating—shifted upward (electron doping) or downward (hole doping) relative to the Dirac point—thereby enabling dynamic modulation of the DOS available for electron transfer [44, 50]. This tunability is particularly advantageous for reactions such as iodide oxidation, where catalytic performance strongly depends on rapid electron exchange between the catalyst and reactants. Moreover, optimizing the alignment of the electrode Fermi level with the electrolyte redox potential can further accelerate reaction kinetics and enhance IOR activity.

To investigate gate‐controlled IOR catalysis on graphene, we fabricated an electrostatic back‐gated device in a field‐effect transistor (FET) configuration and integrated it into the SECCM platform, as illustrated in Figure 2a. A key advantage of SECCM is its compatibility with FET architectures, which enables localized electrochemical measurements on selected nanoscale regions of graphene while isolating the rest of the device from electrolyte‐induced gating and conductivity variations associated with the so‐called self‐gating effect [62]. The FET device was constructed on a highly doped Si substrate serving as a global back gate, separated from the graphene by a 90 nm SiO_2_ dielectric layer. By applying a gate voltage (V_BG_) between the Si back gate and the graphene working electrode, the carrier concentration and Fermi level of the graphene could be continuously tuned across both electron‐ and hole‐doped regimes. The details of graphene FET fabrication are provided in the Supporting Information. Figure 2b shows the I_DS–_V_BG_ curve under ±5 V, exhibiting graphene's typical ambipolar transport behavior and zero‐bandgap signature. The current minimum, corresponding to the charge‐neutrality (Dirac) point, appears to be shifted slightly toward positive gate voltages (∼0.25 V). This slightly p‐doped pristine state, which is mainly attributed to the electron transfer from graphene to environmental oxygen and moisture adsorbates, as well as contributions from polymer or etchant residues introduced during graphene transfer [63, 64, 65, 66], was reproducibly observed in our fabricated devices, as shown in Figure S9. The applied gate voltage effectively modulates the electronic structure of graphene by shifting the Fermi level relative to the Dirac point. For V_BG_ > 0, graphene becomes increasingly n‐type due to electron accumulation, while for V_BG_ < 0, p‐type behavior dominates as the Fermi level shifts downward away from the Dirac point. By using a parallel‐plate capacitor model, the induced carrier concentration in the electrostatically gated monolayer graphene can be quantitatively estimated. Under the experimental conditions (−5 V ≤ V_BG_ ≤ +5 V), the carrier density was estimated to vary from approximately −1.25 × 10^12^ cm^−2^ (hole doping) to +1.14 × 10^12^ cm^−2^ (electron doping), corresponding to Fermi‐level shifts of −0.135 and +0.125 eV relative to the Dirac point, respectively. Detailed calculations of the carrier concentration and Fermi‐level position of the gated graphene FET are provided in the Supporting Information.

**FIGURE 2:**
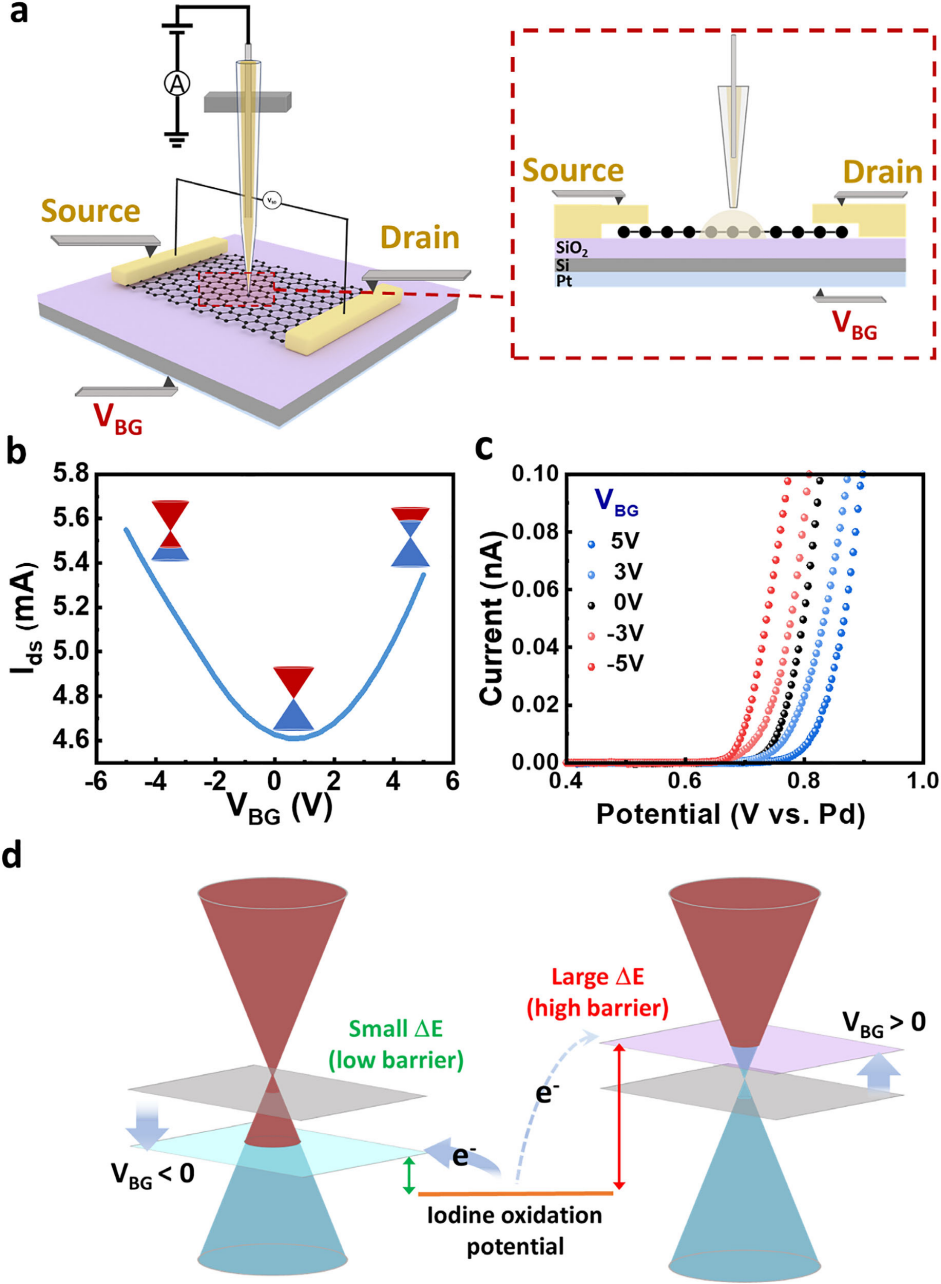
(a) Schematic of operando SECCM setup integrated with back‐gated graphene FET architecture for tuning electronic properties during localized IOR measurements. (b) Transfer characteristics of the graphene transistor under gate bias from −5 to +5 V. The corresponding gate‐dependent SECCM LSV curves are shown in (c). (d) Band diagrams correlating Fermi level shifts with IOR thermodynamics: hole doping (V_BG_ < 0) enhances IOR by lowering the Fermi level toward the redox potential, while electron doping (V_BG_ > 0) suppresses it.

Next, the gate‐induced Fermi‐level shift of graphene was correlated with the evolution of the local IOR response under varying gate voltages, as demonstrated by the representative SECCM LSV curves in Figure 2c. Pristine graphene without gate bias shows an IOR onset potential at 0.75 V vs. Pd. Applying a negative gate bias lowers the onset potential to 0.72 and 0.69 V at −3 and −5 V, respectively, and increases the reaction current, indicative of enhanced catalytic activity and reduced overpotential. In contrast, positive gate biases of +3 and +5 V shift the onset potential to 0.77 and 0.81 V, respectively, and suppress the current response, reflecting reduced catalytic efficiency. This gate‐tunable behavior is consistent with the energetic requirements of iodide oxidation, which depends on efficient electron transfer from iodide ions to the graphene surface. As schematically illustrated in Figure 2d, the redox level of the I^−^/I_3_^−^ couple (+0.54 V vs. SHE or −4.98 eV vs. vacuum) [25] in solution acts as a fixed energy reference, whereas the graphene Fermi level is dynamically tuned by the applied gate voltage. Under negative gate bias, the graphene Fermi level shifts downward (p‐type doping), approaching the I^−^/I_3_^−^ redox level and increasing the population of hole‐like accepting states. This lowers the energetic barrier for electron extraction from iodide and promotes more efficient interfacial charge transfer. In contrast, positive gate bias shifts the Fermi level upward (n‐type doping), which is consistent with a larger electron‐transfer barrier and slower IOR kinetics. The corresponding statistical results are summarized in Figure S14 and Table S1. A Ru(NH_3_)_6_^3+^ reduction control experiment performed under the same gated SECCM graphene platform further supports the modulation of charge transfer energetics. (Figure S18).

While electrostatic gating provides an extrinsic route to regulate the Fermi level in graphene, twist‐angle engineering offers a structurally engineered route to reconfigure the graphene electronic landscape itself [67, 68, 69, 70]. When two graphene sheets are stacked with a small rotational misalignment, the resulting moiré superlattice produces spatially varying AA, AB, and BA stacking environments, accompanied by electronic reconstruction [46, 47, 52, 69, 70]. Near the magic angle (∼1.1°), interlayer hybridization narrows and flattens the low‐energy bands, producing strong DOS enhancement near E_F_ together with highly non‐uniform local electronic weight within the moiré unit cell, where AA‐like regions can carry a disproportionately large contribution [46, 47, 53]. The resulting enhancement of the local DOS has been closely associated with emergent strong‐correlation phenomena, including Mott insulating states and unconventional superconductivity [71, 72]. From an electrochemical perspective, this type of moiré‐engineered interface is relevant not only because it increases the number of states available for interfacial charge transfer, but also because it creates spatially heterogeneous local environments that may influence adsorption, charge‐transfer coupling, and stabilization of iodine‐related species during IOR [46, 47, 54, 55, 56]. We therefore investigate IOR at MATBG by integrating SECCM with a MATBG device. MATBG with a twist angle of ∼1.1° was fabricated by mechanically exfoliated graphene flakes encapsulated in h‐BN using the dry‐transfer tear‐and‐stack method [73] to ensure high device quality (see Supporting Information). Figure 3a presents an atomic schematic of MATBG, illustrating the moiré superlattice resulting from the interlayer twist with a periodicity of ∼13 nm. This periodic structure has a direct counterpart in reciprocal space (Figure 3b), where the interlayer rotation offsets the monolayer K (and K’) points and constructs a moiré mini‐Brillouin zone. Figure 3c displays a representative selected‐area electron diffraction (SAED) pattern from a MATBG specimen prepared by the same fabrication route, where the ∼1.1° splitting between the diffraction spots of the two graphene layers. The twist‐angle homogeneity over several micrometer length scales was confirmed by multi‐position SAED analysis and dual‐gate transport measurements, as provided in the Supporting Information. Figure 3d,e show representative high‐resolution Transmission Electron Microscope (TEM) images of AA and AB stacking regions, respectively, illustrating the distinct local stacking motifs within the moiré superlattice. The strong interlayer hybridization expected near the magic angle is further supported by continuum model calculations [74], as shown in Figure 3f,g. The calculated band structures in a moiré unit cell were overlaid with the spectral weight of the AA and AB stacking regions, respectively. The flat‐band DOS, highlighted in red, is predominantly localized in regions of the moiré unit cell with local AA stacking. Consistently, the projected DOS in Figure 3h,i show a strong contribution from AA regions, while AB regions contribute comparatively little [75].

**FIGURE 3:**
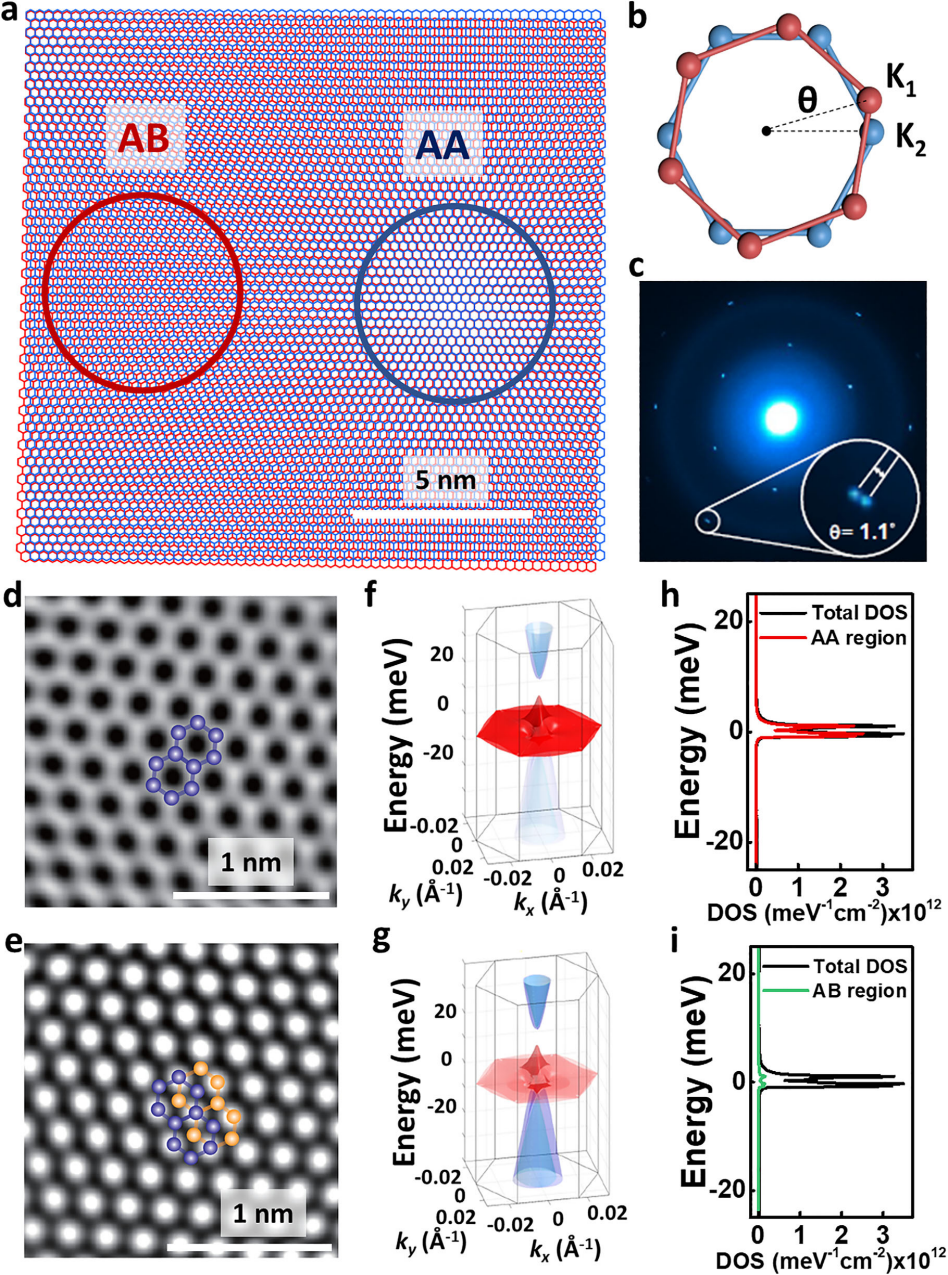
(a) Moiré superlattice of TBG with a twist angle (θ) of 1.1°, exhibiting distinct AA and AB stacking domains. (b) Schematic of Brillouin zones for two graphene layers rotated by an angle *θ*, resulting in the displacement between their Dirac points. (c) SAED pattern confirming the twist angle. (d, e) are TEM images revealing the local stacking configurations of AA and AB stacking regions, respectively. Overlaid atomic schematics highlight the distinct stacking motifs and interlayer registries. (f, g) Calculated Dirac cones of MATBG overlaid with the spectral weight of the AA or AB stacking regions, respectively. (h, i) Projected DOS of AA and AB regions, showing a strong contribution from AA regions, while AB regions contribute comparatively little.

Next, we performed SECCM measurements on various graphene‐based electrodes for IOR, including monolayer graphene, Bernal‐stacked bilayer graphene, and MATBG, all of which were fabricated via mechanical exfoliation. For comparison, the IOR performance of previously prepared CVD‐grown graphene is also included. Figure 4a,b show the schematic architecture and optical image of the SECCM‐compatible MATBG device. The h‐BN/MATBG/h‐BN heterostructure was assembled by dry transfer and contacted in an edge‐contact geometry following established protocols [73, 76, 77]. A lithographically defined square aperture was etched through the top h‐BN to expose the active MATBG region, allowing the SECCM nanopipette to access the surface for localized electrochemical measurements. Complete fabrication details and dimensions are provided in the Supporting Information. Within this windowed architecture, only the exposed MATBG is wetted by the meniscus. Under our SECCM configuration, the nanopipette forms a nanoscopic meniscus with a lateral footprint of ∼100 nm, which is significantly larger than the ∼13 nm moiré superlattice period of MATBG. Consequently, each SECCM pixel spans several AA/AB stacking domains, and each measurement reflects the collective microscopic electrochemical signature that contains ensemble‐averaged, meniscus‐confined response from multiple moiré unit cells rather than a direct electrochemical response of an individual AA or AB domain. Figure 4c presents representative SECCM LSV curves comparing the IOR performance of these devices. MATBG exhibits the strongest IOR response and the lowest onset potential (0.66 V vs. Pd), highlighting its markedly enhanced local electrocatalytic behavior. In contrast, Bernal‐stacked bilayer graphene shows a higher onset potential of 0.71 V vs. Pd. Monolayer graphene, whether obtained via mechanical exfoliation or CVD growth, exhibits similar IOR performance with an onset potential of approximately 0.75 V vs. Pd, while the exfoliated monolayer shows slightly better behavior than the CVD‐grown sample, consistent with its higher crystalline quality. The corresponding statistical results are summarized in Figure S15 and Table S1. To be noted, the SECCM, multi‐position SAED (Figure S10), and dual‐gate transport measurements (Figure S17) were performed on separate MATBG devices fabricated using the same transfer process rather than on the identical device. Therefore, the twist angle of the specific region probed by SECCM was not directly verified. Nevertheless, multi‐position SAED and transport measurements demonstrate micrometer‐scale twist‐angle homogeneity, which is substantially larger than the length scale of the SECCM meniscus footprint. Together with the consistent SECCM responses obtained at multiple locations, these results support the interpretation that the electrochemical behavior is representative of MATBG devices fabricated under the same conditions.

**FIGURE 4:**
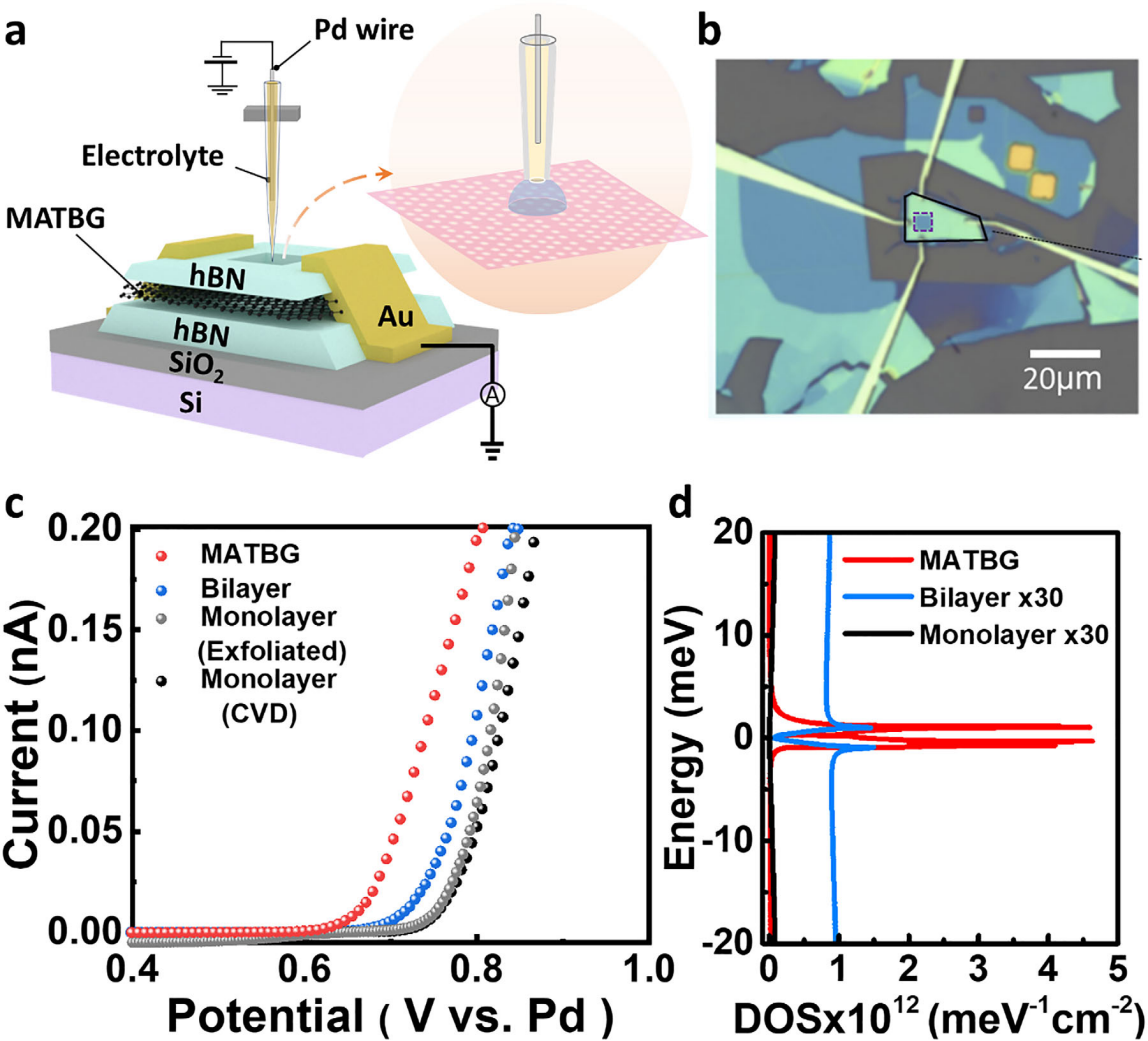
(a) MATBG sample configuration along with a schematic of the electrolyte meniscus on MATBG. The meniscus spans several moiré periods and therefore probes a collective local response from multiple moiré unit cells. (b) Optical image of the MATBG device used for SECCM IOR measurements. (c) Representative SECCM LSV results comparing monolayer graphene, Bernal‐stacked bilayer graphene, CVD‐grown graphene, and MATBG. (d) Calculated DOS of MATBG vs. monolayer and Bernal‐stacked bilayer graphene.

Based on the observed SECCM trends, electronic‐structure calculations, and previous studies on moiré electrochemistry and iodine–graphene interactions [47, 54, 55, 56], the enhanced IOR activity of MATBG can be associated with a reconstructed interfacial electronic environment induced by the magic‐angle moiré superlattice, which may involve the following coupled effects. First, the flat bands increase the electronic‐state density available for interfacial charge transfer (Figure 4d). Second, the spatial localization of these states in AA‐enriched regions creates heterogeneous local electronic environments within the moiré unit cell, which may modify the electronic coupling between graphene and iodine‐related redox species. Third, because iodine adsorption on graphene involves charge‐transfer interactions, the modified local electronic structure of MATBG may influence the adsorption/desorption balance of iodide, iodine, or polyiodide species. In addition, recent studies suggest that enhanced electrode DOS can reduce interfacial reorganization energy through electronic screening. Although these effects cannot be quantitatively separated in the present study, they may act together to promote IOR and are therefore considered a reasonable mechanistic explanation for the enhanced activity of MATBG. Accordingly, MATBG could provide a more optimized interfacial environment for the adsorption and desorption of iodine‐related species to promote efficient IOR compared with monolayer and Bernal‐stacked bilayer graphene.

## 3 Conclusions

In conclusion, this work demonstrates that the iodide oxidation activity of graphene can be effectively regulated through deliberate control of its electronic structure. In monolayer graphene, electrostatic gating provides a direct means to tune the Fermi level and carrier density, thereby modifying the energetic alignment between graphene and the I^−^/I_3_^−^ redox couple and enabling controllable changes in local IOR kinetics. In twisted bilayer graphene, the magic‐angle moiré superlattice introduces a distinct form of electronic‐structure reconstruction, in which flat‐band‐derived states emerge together with spatially varying local stacking environments. Under these conditions, the enhanced IOR response of MATBG is attributed to the combined effects of increased electronic‐state density near the Fermi level and a more favorable interfacial environment for iodine‐related adsorption and charge transfer. By linking nanoscale electrochemical behavior with tunable graphene electronic structures through SECCM, this study provides a coherent framework for understanding how Dirac‐cone engineering can be used to regulate iodine electrochemistry. More broadly, it identifies graphene‐based quantum and moiré engineering as a promising route toward metal‐free catalysts for value‐added anodic reactions.

## Author Contributions

**Yang – Sheng Lu**: methodology, investigation, validation, formal analysis, data curation, Writing – original draft, Writing – review and editing, visualization. **Akichika Kumatani**: methodology, resources. **Kuo – En Chang**: methodology, investigation, formal analysis, validation, data curation, resources. **Kenji Watanabe**: resources. **Ting – Han Lin**: investigation, formal analysis, methodology, resources. **Ming – Hao Liu**: software, resources. **Ming – Wen Chu**: resources, writing – review and editing, writing – original draft, formal analysis, data curation, investigation. **Aitor Garcia – Ruiz**: data curation, formal analysis, investigation, software, methodology, writing – review and editing, writing – original draft. **Ta – Lei Chou**: investigation, formal analysis, data curation, writing – review and editing. **Shao – Sian Li**: conceptualization, writing – original draft, writing – review and editing, funding acquisition, visualization, project administration, data curation, methodology. **Chun – Wei Chen**: conceptualization, supervision, funding acquisition, writing – original draft, writing – review and editing, project administration, resources, methodology. **Takashi Taniguchi**: resources. **Tse – Ming Chen**: conceptualization, project administration, funding acquisition, writing – review and editing, resources, methodology, writing – original draft. **Yen – Ting Liu**: investigation, formal analysis.

## Conflicts of Interest

The authors declare no conflicts of interest.

## Supporting information




**Supporting File**: advs76835‐sup‐0001‐SuppMat.pdf.

## Data Availability

The data that supports the findings of this study are available in the supplementary material of this article.

## References

[1] R. Saini , F. Naaz , A. H. Bashal , A. H. Pandit , and U. Farooq , “Recent Advances In Nitrogen‐Doped Graphene‐Based Heterostructures And Composites: Mechanism And Active Sites For Electrochemical ORR and HER,” Green Chemistry 26, no. 1 (2024): 57–102.

[2] D. M. S. Prado , G. Li , J. A. D. del Rosario , J. D. Ocon , and P. Y. Abel Chuang , “Improved Oxygen Reduction Reaction Activity of Graphene via Mechanochemical Activation and Halogen‐Doping,” ChemElectroChem 12, no. 1 (2025): 202400494.

[3] Y. Jia , L. Zhang , A. Du , et al., “Defect Graphene As A Trifunctional Catalyst For Electrochemical Reactions,” Advanced Materials 28, no. 43 (2016): 9532–9538, 10.1002/adma.201602912. 27622869

[4] H. Zhao , C. Sun , Z. Jin , et al., “Carbon For The Oxygen Reduction Reaction: A Defect Mechanism,” Journal of Materials Chemistry A 3, no. 22 (2015): 11736–11739, 10.1039/C5TA02229K.

[5] Y. Han , X. Yan , Q. Wu , et al., “Defect‐Derived Catalysis Mechanism Of Electrochemical Reactions In Two‐Dimensional Carbon Materials,” Small Structures 4, no. 10 (2023): 2300036.

[6] H. Zheng , Y. Lu , K.‐H. Ye , et al., “Atomically Thin Photoanode Of InSe/Graphene Heterostructure,” Nature Communications 12, no. 1 (2021): 91. 10.1038/s41467-020-20341-7 PMC7782821 33398029

[7] H. Gu , L. Zhong , G. Shi , et al., “Graphdiyne/Graphene Heterostructure: A Universal 2D Scaffold Anchoring Monodispersed Transition‐Metal Phthalocyanines For Selective And Durable CO_2_ Electroreduction,” Journal of the American Chemical Society 143, no. 23 (2021): 8679–8688. 10.1021/jacs.1c02326 34077183

[8] V. Thirumal , R. Yuvakkumar , P. S. Kumar , et al., “Heterostructured Two Dimensional Materials Of MXene And Graphene By Hydrothermal Method For Efficient Hydrogen Production And HER Activities,” International Journal of Hydrogen Energy 48, no. 17 (2023): 6478–6487.

[9] J. Wang , M. Yan , K. Zhao , et al., “Field Effect Enhanced Hydrogen Evolution Reaction Of MoS_2_ Nanosheets,” Advanced Materials 29, no. 7 (2017): 1604464, 10.1002/adma.201604464. 27966252

[10] Y. Wang , S. Udyavara , M. Neurock , and C. D. Frisbie , “Field Effect Modulation Of Electrocatalytic Hydrogen Evolution At Back‐Gated Two‐Dimensional MoS_2_ Electrodes,” Nano Letters 19, no. 9 (2019): 6118–6123, 10.1021/acs.nanolett.9b02079. 31434483

[11] Y. Pan , X. Wang , W. Zhang , et al., “Boosting The Performance Of Single‐Atom Catalysts Via External Electric Field Polarization,” Nature Communications 13, no. 1 (2022): 3063. 10.1038/s41467-022-30766-x PMC9163078 35654804

[12] Z. Peng , X. Chen , Y. Fan , D. J. Srolovitz , and D. Lei , “Strain Engineering Of 2D Semiconductors And Graphene: From Strain Fields To Band‐Structure Tuning And Photonic Applications,” Light: Science & Applications 9, no. 1 (2020): 190. 10.1038/s41377-020-00421-5 PMC7680797 33298826

[13] A. Chaves , J. G. Azadani , H. Alsalman , et al., “Bandgap Engineering Of Two‐Dimensional Semiconductor Materials,” npj 2D Materials and Applications 4, no. 1 (2020): 29, 10.1038/s41699-020-00162-4.

[14] H. Zhu , Y. Liu , Y. Wu , Y. He , Y. Cao , and S. Hu , “Electrocatalytic Stability Of Two‐Dimensional Materials,” Journal of Energy Chemistry 97 (2024): 302–320, 10.1016/j.jechem.2024.05.044.

[15] D. Voiry , A. Mohite , and M. Chhowalla , “Phase Engineering Of Transition Metal Dichalcogenides,” Chemical Society Reviews 44, no. 9 (2015): 2702–2712, 10.1039/C5CS00151J. 25891172

[16] E. A. Moges , C.‐Y. Chang , M.‐C. Tsai , W.‐N. Su , and B. J. Hwang , “Electrocatalysts For Value‐Added Electrolysis Coupled With Hydrogen Evolution,” EES Catalysis 1, no. 4 (2023): 413–433.

[17] E. A. Moges , K. Lakshmanan , C.‐Y. Chang , et al., “Materials Of Value‐Added Electrolysis For Green Hydrogen Production,” ACS Materials Letters 6, no. 11 (2024): 4932–4954.

[18] C. Rong , X. Huang , H. Arandiyan , Z. Shao , Y. Wang , and Y. Chen , “Advances In Oxygen Evolution Reaction Electrocatalysts Via Direct Oxygen–Oxygen Radical Coupling Pathway,” Advanced Materials 37, no. 9 (2025): 2416362. 10.1002/adma.202416362 PMC11881674 39815381

[19] Y. Yao , J. Lyu , X. Li , et al., “A Review Of Efficient Electrocatalysts For The Oxygen Evolution Reaction At Large Current Density,” DeCarbon 5 (2024): 100062, 10.1016/j.decarb.2024.100062.

[20] S. Wang , T. He , J. H. Yun , et al., “New Iron‐Cobalt Oxide Catalysts Promoting BiVO_4_ Films for Photoelectrochemical Water Splitting,” Advanced Functional Materials 28, no. 34 (2018): 1802685, 10.1002/adfm.201802685.

[21] G. Dong , L. Yan , and Y. Bi , “Advanced Oxygen Evolution Reaction Catalysts For Solar‐Driven Photoelectrochemical Water Splitting,” Journal of Materials Chemistry A 11, no. 8 (2023): 3888–3903, 10.1039/D2TA09479G.

[22] S.‐M. Peng , S. B. Patil , C.‐C. Chang , et al., “Fast Charge Transfer Between Iodide Ions And A Delocalized Electron System On The Graphite Surface For Boosting Hydrogen Production,” Journal of Materials Chemistry A 10, no. 45 (2022): 23982–23989, 10.1039/D2TA06517G.

[23] T. A. Dessie , W.‐H. Huang , D. B. Adam , et al., “Efficient H_2_ Evolution Coupled With Anodic Oxidation Of Iodide Over Defective Carbon‐Supported Single‐Atom Mo‐N_4_ Electrocatalyst,” Nano Letters 22, no. 18 (2022): 7311–7317. 10.1021/acs.nanolett.2c01229 36107720

[24] Y. S. Park , X. Jin , J. Tan , et al., “High‐Performance Sb_2_S_3_ Photoanode Enabling Iodide Oxidation Reaction For Unbiased Photoelectrochemical Solar Fuel Production,” Energy & Environmental Science 15, no. 11 (2022): 4725–4737.

[25] S. Trasatti , “The Absolute Electrode Potential: An Explanatory Note (Recommendations 1986),” Pure and Applied Chemistry 58, no. 7 (1986): 955–966, 10.1351/pac198658070955.

[26] L. Ma , G. Zhu , Z. Wang , et al., “Long‐Lasting Zinc–Iodine Batteries With Ultrahigh Areal Capacity And Boosted Rate Capability Enabled By Nickel Single‐Atom Electrocatalysts,” Nano Letters 23, no. 11 (2023): 5272–5280. 10.1021/acs.nanolett.3c01310 37260235

[27] K. Zhang , Q. Yu , J. Sun , Z. Tie , and Z. Jin , “Precipitated Iodine Cathode Enabled by Trifluoromethanesulfonate Oxidation for Cathode/Electrolyte Mutualistic Aqueous Zn–I Batteries,” Advanced Materials 36, no. 6 (2024): 2309838. 10.1002/adma.202309838 37949441

[28] L. She , H. Cheng , Z. Yuan , et al., “Rechargeable Aqueous Zinc–Halogen Batteries: Fundamental Mechanisms, Research Issues, And Future Perspectives,” Advanced Science 11, no. 8 (2024): 2305061. 10.1002/advs.202305061 PMC10953720 37939285

[29] K. Zhang and Z. Jin , “Halogen‐Enabled Rechargeable Batteries: Current Advances And Future Perspectives,” Energy Storage Materials 45 (2022): 332–369, 10.1016/j.ensm.2021.11.048.

[30] N. Naresh , S. N. Paltasingh , Y. Zhu , et al., “3D Engineered Dual‐Redox Zinc‐Iodine Microbatteries for Intrinsically Safe on‐Chip Energy Storage,” Advanced Science (2026): 75453, 10.1002/advs.75453. PMC13335492 42043786

[31] K. Zhang , Q. Yu , J. Sun , et al., “Halogen Electrochemistry Enabled Complementary Lithium/Halogen Dual‐Ion Batteries With Enhanced Capacity and Cyclability,” Nano Letters 25, no. 46 (2025): 16554–16563. 10.1021/acs.nanolett.5c04968 41194427

[32] J.‐J. Zeng , Z.‐X. Wang , and F.‐G. Wu , “Iodine‐Containing Materials For Biomedical Applications,” Journal of Materials Chemistry B 13 (2025): 8583–8597. 10.1039/d5tb00534e 40566879

[33] Y. Takahashi , Y. Kobayashi , Z. Wang , et al., “High‐Resolution Electrochemical Mapping of the Hydrogen Evolution Reaction on Transition‐Metal Dichalcogenide Nanosheets,” Angewandte Chemie International Edition 59, no. 9 (2020): 3601–3608, 10.1002/anie.201912863. 31777142

[34] D. Rafalskyi , J. M. Martínez , L. Habl , et al., “In‐Orbit Demonstration Of An Iodine Electric Propulsion System,” Nature 599, no. 7885 (2021): 411–415. 10.1038/s41586-021-04015-y PMC8599014 34789903

[35] P. L. Bigliardi , S. A. L. Alsagoff , H. Y. El‐Kafrawi , J.‐K. Pyon , C. T. C. Wa , and M. A. Villa , “Povidone Iodine In Wound Healing: A Review Of Current Concepts And Practices,” International Journal of Surgery 44 (2017): 260–268, 10.1016/j.ijsu.2017.06.073. 28648795

[36] D. B. Adam , M.‐C. Tsai , Y. A. Awoke , et al., “Engineering Self‐Supported Ruthenium‐Titanium Alloy Oxide On 3d Web‐Like Titania As Iodide Oxidation Reaction Electrocatalyst To Boost Hydrogen Production,” Applied Catalysis B: Environmental 316 (2022): 121608, 10.1016/j.apcatb.2022.121608.

[37] D. B. Adam , M.‐C. Tsai , Y. A. Awoke , et al., “Iodide Oxidation Reaction Catalyzed By Ruthenium–Tin Surface Alloy Oxide For Efficient Production Of Hydrogen And Iodine Simultaneously,” ACS Sustainable Chemistry & Engineering 9, no. 26 (2021): 8803–8812.

[38] Y. S. Park , G. Jang , I. Sohn , et al., “Efficient Solar Fuel Production Enabled By An Iodide Oxidation Reaction On Atomic Layer Deposited MoS_2_ ,” Carbon Energy 5, no. 12 (2023): 366.

[39] S. Paniya and K. Vankayala , “Enhanced H_2_ Production Assisted By Anodic Iodide Oxidation Using Transparent Tin Oxide‐Based Electrodes,” Chemical Communications 60, no. 56 (2024): 7208–7211. 10.1039/d4cc01717j 38910534

[40] S. Zhao , T. Liu , Y. Dai , et al., “Pt/C As A Bifunctional ORR/Iodide Oxidation Reaction (IOR) Catalyst for Zn‐air Batteries With Unprecedentedly High Energy Efficiency of 76.5%,” Applied Catalysis B: Environmental 320 (2023): 121992, 10.1016/j.apcatb.2022.121992.

[41] A. K. Geim and K. S. Novoselov , “The Rise Of Graphene,” Nature Materials 6, no. 3 (2007): 183–191, 10.1038/nmat1849. 17330084

[42] X. Shen , Y. Liu , Y. Pang , and W. Yao , “Conjugation Of Graphene On Au Surface By π–π Interaction And Click Chemistry,” Electrochemistry Communications 30 (2013): 13–16, 10.1016/j.elecom.2013.01.025.

[43] C. Hébert , E. Masvidal‐Codina , A. Suarez‐Perez , et al., “Flexible Graphene Solution‐Gated Field‐Effect Transistors: Efficient Transducers For Micro‐Electrocorticography,” Advanced Functional Materials 28, no. 12 (2018): 1703976.

[44] H. Xu , Y. Chen , J. Zhang , and H. Zhang , “Investigating The Mechanism Of Hysteresis Effect In Graphene Electrical Field Device Fabricated On SiO_2_ Substrates Using Raman Spectroscopy,” Small 8, no. 18 (2012): 2833–2840. 10.1002/smll.201102468 22678822

[45] A. V. Klekachev , A. Nourbakhsh , I. Asselberghs , A. L. Stesmans , M. M. Heyns , and S. De Gendt , “Graphene Transistors And Photodetectors,” Interface Magazine 22, no. 1 (2013): 63–68, 10.1149/2.F07131if.

[46] M. Van Winkle , K. Zhang , and D. K. Bediako , “Nanoscale Structure And Interfacial Electrochemical Reactivity Of Moiré‐Engineered Atomic Layers,” Accounts of Chemical Research 58, no. 3 (2025): 415–427. 10.1021/acs.accounts.4c00692 39817845

[47] Y. Yu , K. Zhang , H. Parks , et al., “Tunable Angle‐Dependent Electrochemistry At Twisted Bilayer Graphene With Moiré Flat Bands,” Nature Chemistry 14, no. 3 (2022): 267–273. 10.1038/s41557-021-00865-1 35177786

[48] M. A. Bissett , S. Konabe , S. Okada , M. Tsuji , and H. Ago , “Enhanced Chemical Reactivity Of Graphene Induced By Mechanical Strain,” ACS Nano 7, no. 11 (2013): 10335–10343, 10.1021/nn404746h. 24131427

[49] H. J. Lee , J. Hwang , H. C. Hong , et al., “Electrochemical Reactivity Of Graphene Under Mechanical Strain,” Journal of Materials Chemistry A 13, no. 29 (2025): 23696–23705, 10.1039/D5TA02062J.

[50] L. Zheng , X. Cheng , Z. Wang , et al., “Reversible n‐type Doping Of Graphene by H_2_O‐Based Atomic‐Layer Deposition And Its Doping Mechanism,” The Journal of Physical Chemistry C 119, no. 11 (2015): 5995–6000, 10.1021/jp511562t.

[51] G. Abbas , F. J. Sonia , M. Jindra , et al., “Electrostatic Gating Of Monolayer Graphene By Concentrated Aqueous Electrolytes,” The Journal of Physical Chemistry Letters 14, no. 18 (2023): 4281–4288. 10.1021/acs.jpclett.3c00814 PMC10184166 37126786

[52] S. H. Sung , Y. M. Goh , H. Yoo , et al., “Torsional Periodic Lattice Distortions And Diffraction Of Twisted 2D Materials,” Nature Communications 13, no. 1 (2022): 7826. 10.1038/s41467-022-35477-x PMC9763474 36535920

[53] Z. Wang , F. Liu , and M. Chou , “Fractal Landau‐Level Spectra In Twisted Bilayer Graphene,” Nano Letters 12, no. 7 (2012): 3833–3838, 10.1021/nl301794t. 22716657

[54] S. Maroo , L. Coello Escalante , Y. Wang , et al., “Electronic Origin Of Reorganization Energy In Interfacial Electron Transfer,” Nature 653 (2026): 98–103, 10.1038/s41586-026-10311-2. PMC13149326 42020754

[55] R. A. Dziatko , B. E. Janicek , J. L. Karten , K. A. Harris , M. M. Gibson , and A. C. Crowther , “Solvent‐Mediated Chemical Hole Doping of Graphene by Iodine,” The Journal of Physical Chemistry C 124, no. 6 (2020): 3827–3834, 10.1021/acs.jpcc.9b09182.

[56] R. A. Hoyt , E. M. Remillard , E. D. Cubuk , C. D. Vecitis , and E. Kaxiras , “Polyiodide‐Doped Graphene,” The Journal of Physical Chemistry C 121, no. 1 (2017): 609–615, 10.1021/acs.jpcc.6b11653.

[57] N. Ebejer , A. G. Güell , S. C. Lai , K. McKelvey , M. E. Snowden , and P. R. Unwin , “Scanning Electrochemical Cell Microscopy: A Versatile Technique For Nanoscale Electrochemistry And Functional Imaging,” Annual Review of Analytical Chemistry 6, no. 1 (2013): 329–351, 10.1146/annurev-anchem-062012-092650. 23560932

[58] B. Tao , P. R. Unwin , and C. L. Bentley , “Nanoscale Variations In The Electrocatalytic Activity Of Layered Transition‐Metal Dichalcogenides,” The Journal of Physical Chemistry C 124, no. 1 (2019): 789–798, 10.1021/acs.jpcc.9b10279.

[59] H. Xia , X. Sang , Z. Shu , et al., “The Practice Of Reaction Window In An Electrocatalytic On‐Chip Microcell,” Nature Communications 14, no. 1 (2023): 6838. 10.1038/s41467-023-42645-0 PMC10611802 37891203

[60] M. Yan , X. Pan , P. Wang , et al., “Field‐Effect Tuned Adsorption Dynamics of VSe_2_ Nanosheets For Enhanced Hydrogen Evolution Reaction,” Nano Letters 17, no. 7 (2017): 4109–4115, 10.1021/acs.nanolett.7b00855. 28585826

[61] J. Wang , M. Qiu , Y. Jiang , et al., “Emerging On‐Chip Microcells In Electrocatalysis: Functions Of Window And Circuit,” EES Catalysis 1, no. 6 (2023): 874–891.

[62] Y. He , Q. He , L. Wang , et al., “Self‐Gating In Semiconductor Electrocatalysis,” Nature Materials 18, no. 10 (2019): 1098–1104, 10.1038/s41563-019-0426-0. 31332336

[63] C. Melios , A. Centeno , A. Zurutuza , et al., “Effects Of Humidity On The Electronic Properties Of Graphene Prepared By Chemical Vapour Deposition,” Carbon 103 (2016): 273–280, 10.1016/j.carbon.2016.03.018.

[64] P. Wei , N. Liu , H. R. Lee , et al., “Tuning the Dirac Point in CVD‐grown Graphene Through Solution Processed N‐Type Doping With 2‐(2‐methoxyphenyl)‐1, 3‐dimethyl‐2, 3‐dihydro‐1 H‐benzoimidazole,” Nano letters 13, no. 5 (2013): 1890–1897, 10.1021/nl303410g. 23537351

[65] W. Zhang , C.‐T. Lin , K.‐K. Liu , et al., “Opening An Electrical Band Gap Of Bilayer Graphene With Molecular Doping,” ACS Nano 5, no. 9 (2011): 7517–7524, 10.1021/nn202463g. 21819152

[66] D. H. Tien , J.‐Y. Park , K. B. Kim , et al., “Study Of Graphene‐Based 2D‐Heterostructure Device Fabricated By All‐Dry Transfer Process,” ACS Applied Materials & Interfaces 8, no. 5 (2016): 3072–3078, 10.1021/acsami.5b10370. 26771834

[67] A. Nimbalkar and H. Kim , “Opportunities And Challenges In Twisted Bilayer Graphene: A Review,” Nano‐Micro Letters 12, no. 1 (2020): 126, 10.1007/s40820-020-00464-8. PMC7770697 34138115

[68] J. Wang , X. Mu , L. Wang , and M. Sun , “Properties And Applications Of New Superlattice: Twisted Bilayer Graphene,” Materials Today Physics 9 (2019): 100099, 10.1016/j.mtphys.2019.100099.

[69] E. Y. Andrei and A. H. MacDonald , “Graphene Bilayers With A Twist,” Nature Materials 19, no. 12 (2020): 1265–1275, 10.1038/s41563-020-00840-0. 33208935

[70] Y. Cao , V. Fatemi , A. Demir , et al., “Correlated Insulator Behaviour At Half‐Filling In Magic‐Angle Graphene Superlattices,” Nature 556, no. 7699 (2018): 80–84, 10.1038/nature26154. 29512654

[71] K. P. Nuckolls , M. Oh , D. Wong , et al., “Strongly Correlated Chern Insulators In Magic‐Angle Twisted Bilayer Graphene,” Nature 588, no. 7839 (2020): 610–615. 10.1038/s41586-020-3028-8 33318688

[72] Y. Cao , V. Fatemi , S. Fang , et al., “Unconventional Superconductivity In Magic‐Angle Graphene Superlattices,” Nature 556, no. 7699 (2018): 43–50, 10.1038/nature26160. 29512651

[73] L. Wang , I. Meric , P. Huang , et al., “One‐Dimensional Electrical Contact To A Two‐Dimensional Material,” Science 342, no. 6158 (2013): 614–617, 10.1126/science.1244358. 24179223

[74] A. Garcia‐Ruiz , H.‐Y. Deng , V. V. Enaldiev , and V. I. Fal'ko , “Full Slonczewski‐Weiss‐McClure Parametrization Of Few‐Layer Twistronic Graphene,” Physical Review B 104, no. 8 (2021): 085402.

[75] Y. Choi , J. Kemmer , Y. Peng , et al., “Electronic Correlations In Twisted Bilayer Graphene Near The Magic Angle,” Nature Physics 15, no. 11 (2019): 1174–1180, 10.1038/s41567-019-0606-5.

[76] Y. Cao , J. Luo , V. Fatemi , et al., “Superlattice‐Induced Insulating States And Valley‐Protected Orbits In Twisted Bilayer Graphene,” Physical Review Letters 117, no. 11 (2016): 116804, 10.1103/PhysRevLett.117.116804. 27661712

[77] K. Kim , M. Yankowitz , B. Fallahazad , et al., “Van Der Waals Heterostructures With High Accuracy Rotational Alignment,” Nano Letters 16, no. 3 (2016): 1989–1995, 10.1021/acs.nanolett.5b05263. 26859527

