# Supplementary material for: Engineering Dirac Cones in Graphene for Efficient Iodide Catalysis: Insights by Nanoscale Electrochemistry
Source: Adv Sci (Weinh). 2026 Aug 10;13(59):e76835. doi: 10.1002/advs.76835 (PMC13454972; doi:10.1002/advs.76835)
Supplement: Supplementary file 1 [file ADVS-13-e76835-s001.pdf]

Supporting Information

Engineering Dirac Cones in Graphene for Efficient Iodide Catalysis: Insights By Nanoscale Electrochemistry

Author

Yang-Sheng Lu[1,2], Kuo-En Chang[3, 4], Ta-Lei Chou[5], Aitor Garcia-Ruiz[3, 4], Ting-Han Lin[3], Yen-Ting Liu[3], Akichika Kumatani[6, 7], Ming-Hao Liu[3, 4], Kenji Watanabe[8], Takashi Taniguchi[9], Ming-Wen Chu[2, 5], Tse-Ming Chen*[3, 4], Chun-Wei Chen*[2, 5, 10], and Shao-Sian Li*[1]

Affiliation

1. Department of Materials and Mineral Resources Engineering, National Taipei University of Technology, Taipei 10608, Taiwan
2. Center of Atomic Initiative for New Materials, National Taiwan University, Taipei 10617, Taiwan
3. Department of Physics, National Cheng Kung University, Tainan 701, Taiwan
4. Center for Quantum Frontiers of Research & Technology (QFort), National Cheng Kung University, Tainan 701, Taiwan
5. Center for Condensed Matter Sciences, National Taiwan University, Taipei 10617, Taiwan
6. Department of Electrical and Electronic Engineering, Chiba Institute of Technology, Chiba 275-0016, Japan
7. WPI-Advanced Institute of Materials Research and Graduate School of Engineering, Tohoku University, Sendai 980-8577, Japan
8. Research Center for Electronic and Optical Materials, National Institute for Materials Science, Namiki 1-1, Tsukuba, 305-0044, Ibaraki, Japan
9. Research Center for Materials Nanoarchitectonics, National Institute for Materials Science, Namiki 1-1, Tsukuba, 305-0044, Ibaraki, Japan
10. Department of Materials Science and Engineering, National Taiwan University, Taipei 10617, Taiwan

1. Graphene Characterization

Graphene grown on copper foil was transferred onto a Si/SiO_2 substrate using a wet chemical method with poly (ethylene-co-vinyl acetate) (EVA). EVA was spin-coated onto the graphene-covered copper foil at 3500 rpm for 60 s and baked at 90 °C for 10 min to form a protective layer. The EVA-coated graphene/copper foil was then floated in an aqueous ferric chloride solution to etch away the copper. After complete etching, the EVA–graphene membrane was carefully scooped out and rinsed multiple times with deionized water to remove any residual etchant. The membrane was subsequently transferred onto the Si/SiO_2 substrate and baked under vacuum to improve the adhesion of graphene to the substrate. Finally, the EVA layer was removed by dissolving it in hot dimethylbenzene (o-xylene), leaving a clean graphene film on the substrate.

The transferred graphene was comprehensively characterized using Raman spectroscopy, atomic force microscopy (AFM), and optical microscopy to assess its structural quality and morphology. Raman spectra were collected on both the basal plane and edges of the graphene using a 532 nm laser with a power of 50mW and an integration time of 10 s (Figure S1). On the basal plane, the characteristic G (~1580 cm^{-1}) and 2D (~2680 cm^{-1}) peaks were clearly observed, with an intensity ratio I_{2D}/I_G of 2.2 and a 2D peak FWHM of 38 cm^{-1}, consistent with single graphene. The D peak (~1350 cm^{-1}), indicative of defects, exhibited an intensity of I_D/I_G of 0.1, suggesting low defect density. In contrast, spectra collected at the edges showed a relatively enhanced D peak intensity (I_D/I_G of 0.5), indicating higher defect density, while the G and 2D peaks appeared at ~1580 cm^{-1} and ~2696 cm^{-1}, with an I_{2D}/I_G ratio of 0.67 and 2D peak FWHM of 63 cm^{-1}. These results confirm that the basal plane maintains high crystallinity and minimal defects, whereas the edges exhibit slightly increased disorder, in agreement with typical CVD-grown monolayer graphene.

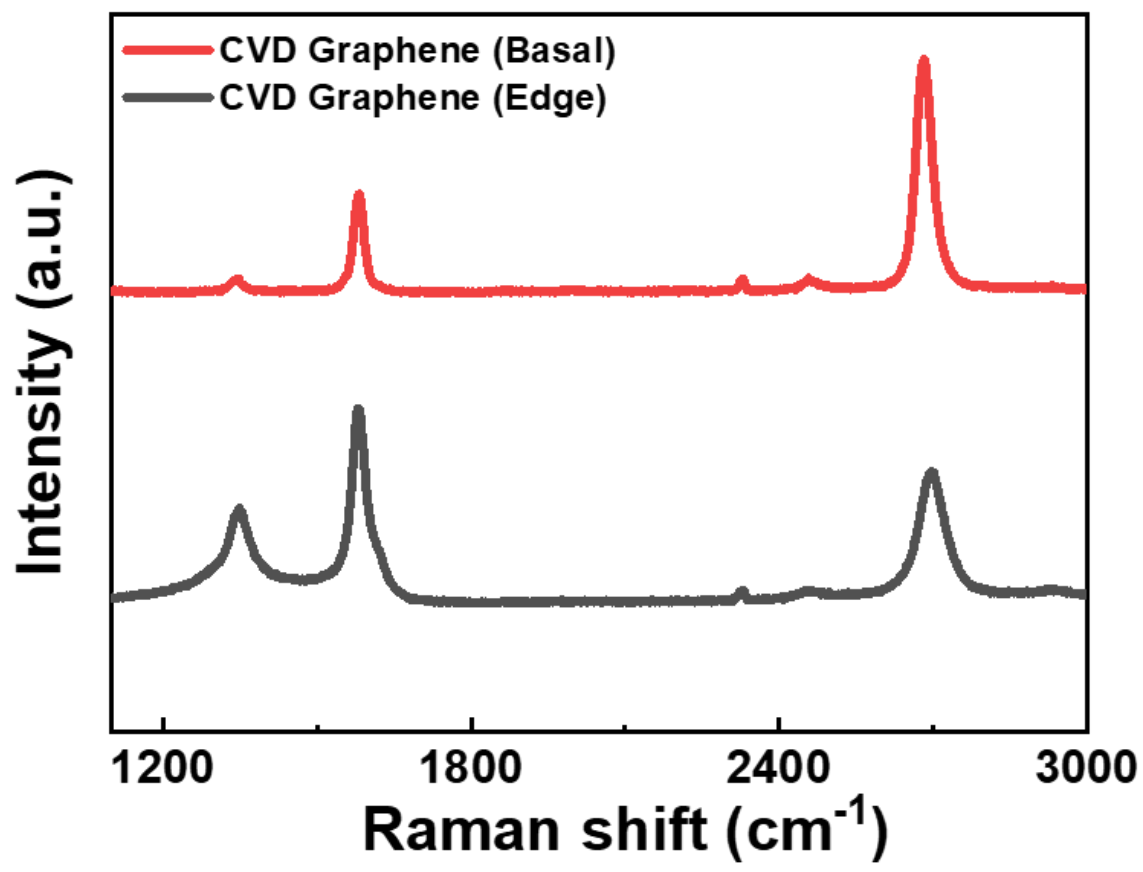

Figure S1. Raman spectra of monolayer graphene at an excitation wavelength of 532nm

AFM was employed to examine the surface topography and thickness of the transferred graphene (Figure S2). The images revealed a continuous and uniform graphene layer without noticeable tears or folds. Height profile analysis indicated an average thickness of approximately 0.83 nm, consistent with monolayer graphene, further confirming the uniformity and smoothness of the film across the substrate.

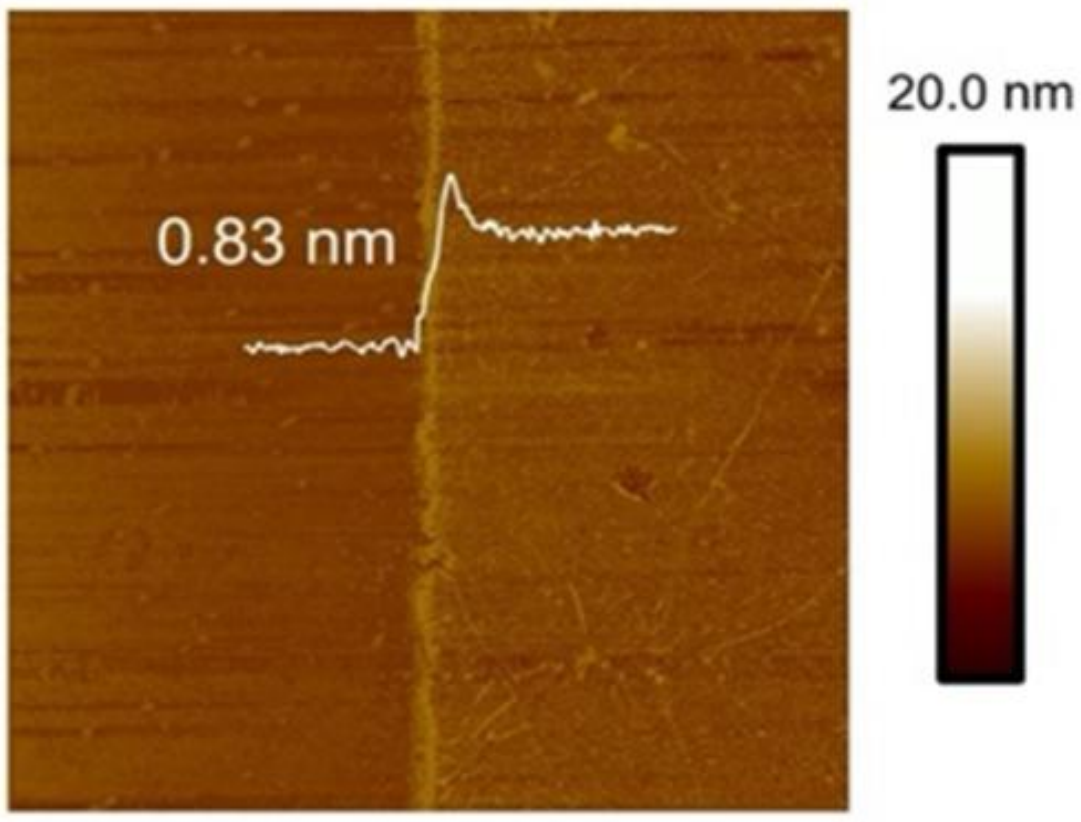

Figure S2. AFM image of CVD-grown single layer graphene on a silicon wafer.

Optical microscopy was used to evaluate the macroscopic coverage of graphene on the n^+ Si/SiO_2 substrate (Figure S3). The image demonstrated continuous graphene regions with minimal visible defects or discontinuities. The contrast between graphene and the substrate allowed clear identification of monolayer coverage, confirming successful transfer and uniform adhesion of the graphene film onto the substrate.

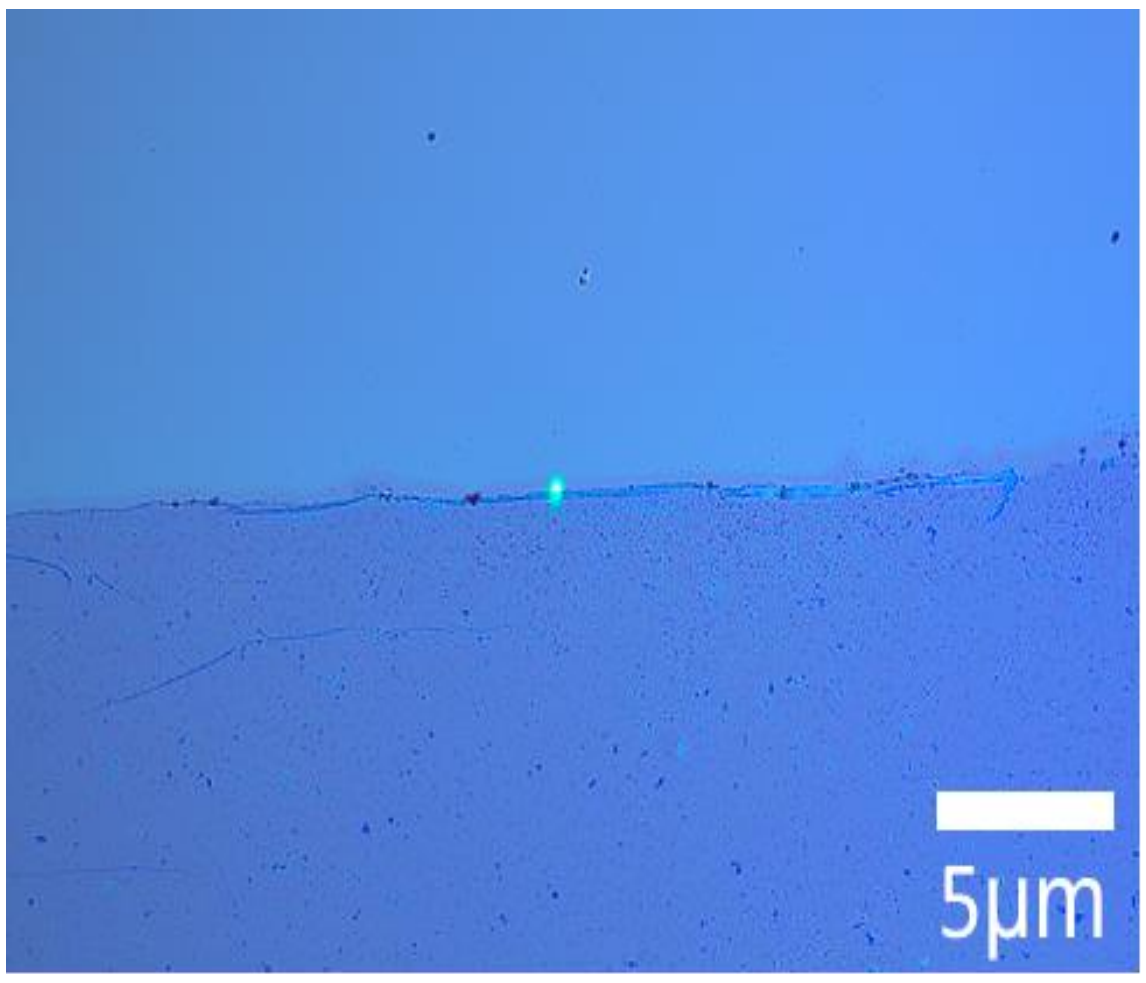

Figure S3. OM image of the as-grown graphene single crystal transferred onto the n^+ Si/SiO_2substrate

2. Macroscale IOR reaction

The macroscale IOR was carried out on graphene/n$^+$-Si using a conventional three-electrode configuration, with a saturated Ag/AgCl electrode as the reference and a Pt wire as the counter electrode. The working electrode had an exposed geometric area of 0.25 cm^2. Linear sweep voltammetry (LSV, 100 mV s^{-1}) and Tafel analysis were performed in 0.5M H_2SO_4 + 0.1 M NaI electrolyte. These bulk measurements reflect the averaged electrode response. Representative LSV and Tafel plots are shown in Figure S4.

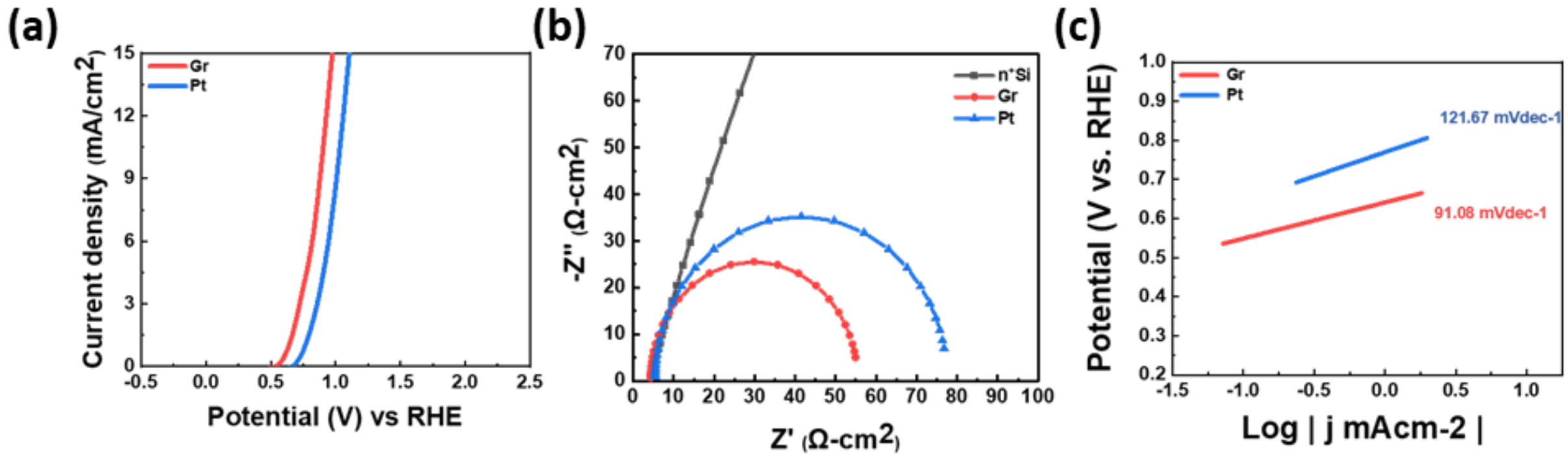

Figure S4. Macroscale electrochemical characterization of transferred graphene on n$^+$ Si. (a) Linear sweep voltammograms (LSVs) measured in 0.5M H_2SO_4 + 0.1 M NaI at 100 mVs^{-1}. (b) Electrochemical impedance spectroscopy (EIS) Nyquist plots showing the interfacial charge transfer behavior. (c) Tafel plots derived from the LSVs, illustrating the reaction kinetics of iodide oxidation.

3. SECCM measurement parameters

All nanoscale electrochemical experiments were conducted using a scanning electrochemical cell microscopy (SECCM) system. Single-barreled nanopipettes (~100 nm diameter, as shown in the SEM image of Figure S5) were filled with an electrolyte solution (50 mM H_2SO_4 + 0.1 M NaI) and contained a Pd wire (diameter ~250 nm) as a quasi-reference counter electrode (QRCE). The graphene-based working electrodes were mounted on an xy-piezoelectric stage, while the pipette probe was controlled by a z-piezoelectric positioner.

The probe was approached toward the graphene surface a constant rate of 20 nm/ms until meniscus contact was established, which was detected through the appearance of a small surface current (about 2 pA). This meniscus-defined junction confined the electrochemical reaction to a nanoscale area without direct contact between the glass pipette and the sample surface. After contact formation, a short stabilization period of 20 ms was allowed before the local voltammetric acquisition of IOR was performed at each predefined position in a hopping-mode grid. The final z-position at each landing was also used to generate a topographical map

of the electrode surface.

LSV was conducted at a scan rate of 100 mVs^{-1}, with a measurement time of 20 ms per point. Typical imaging of a 10 μm × 10 μm area at 64 × 64 pixels required ~30 min. IOR current maps were obtained at an applied potential of 0.85 V versus the Pd QRCE. This protocol was applied to CVD-grown monolayer graphene, mechanically exfoliated monolayer graphene, Bernal-stacked bilayer graphene, and twisted bilayer graphene, enabling direct comparison of local electrocatalytic activity across different graphene systems.

Keep maintain the consistency of measurements and avoid the IOR deviation caused by the pipette geometry and meniscus size, a pre-screen of nanopipette was carried out by measuring IOR on Au substrate. As in Figure S5, showing IOR responses on Au using various nanopipette P1 ~ P5, only probes showing comparable IOR responses (P1, P2 and P3) were used for the subsequent graphene experiments, whereas probes showing large deviations (P4 and P5) were excluded. This screening step was introduced specifically to reduce probe-to-probe variation arising from differences in pipette geometry or effective meniscus response. Therefore, the measured IOR responses more reliably reflect the intrinsic differences among the investigated materials under consistent SECCM conditions.

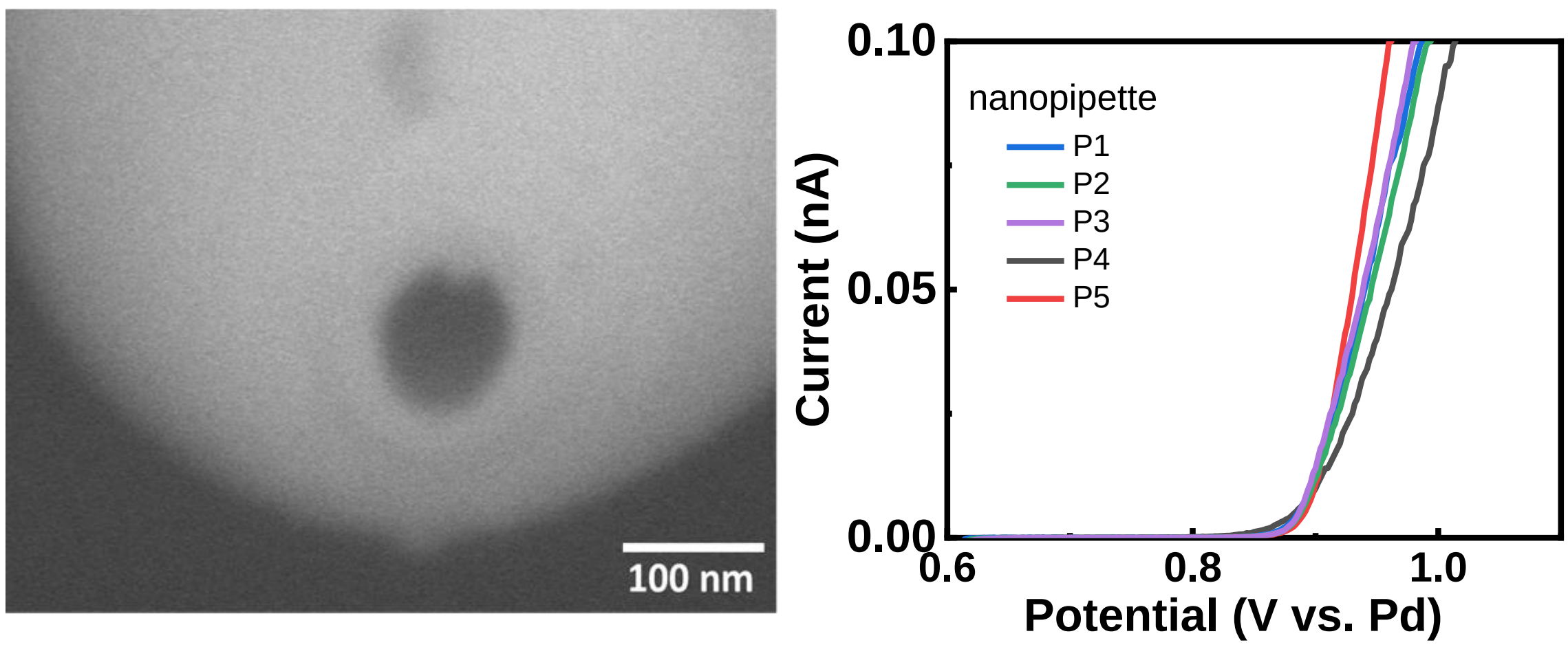

Figure S5. SEM image of nanopipette tip and IOR response of various nanopipette on Au substrate.

4. IOR and OER using SECCM

Figure S6 shows the IOR and OER LSV curves of graphene, conducted in 0.1 M NaI + 50 mM H_2SO_4 (IOR condition) and 50 mM H_2SO_4 only (OER condition) using SECCM,

respectively. The IOR occurs at lower potentials than OER, indicating a preferential activity of graphene toward iodide oxidation.

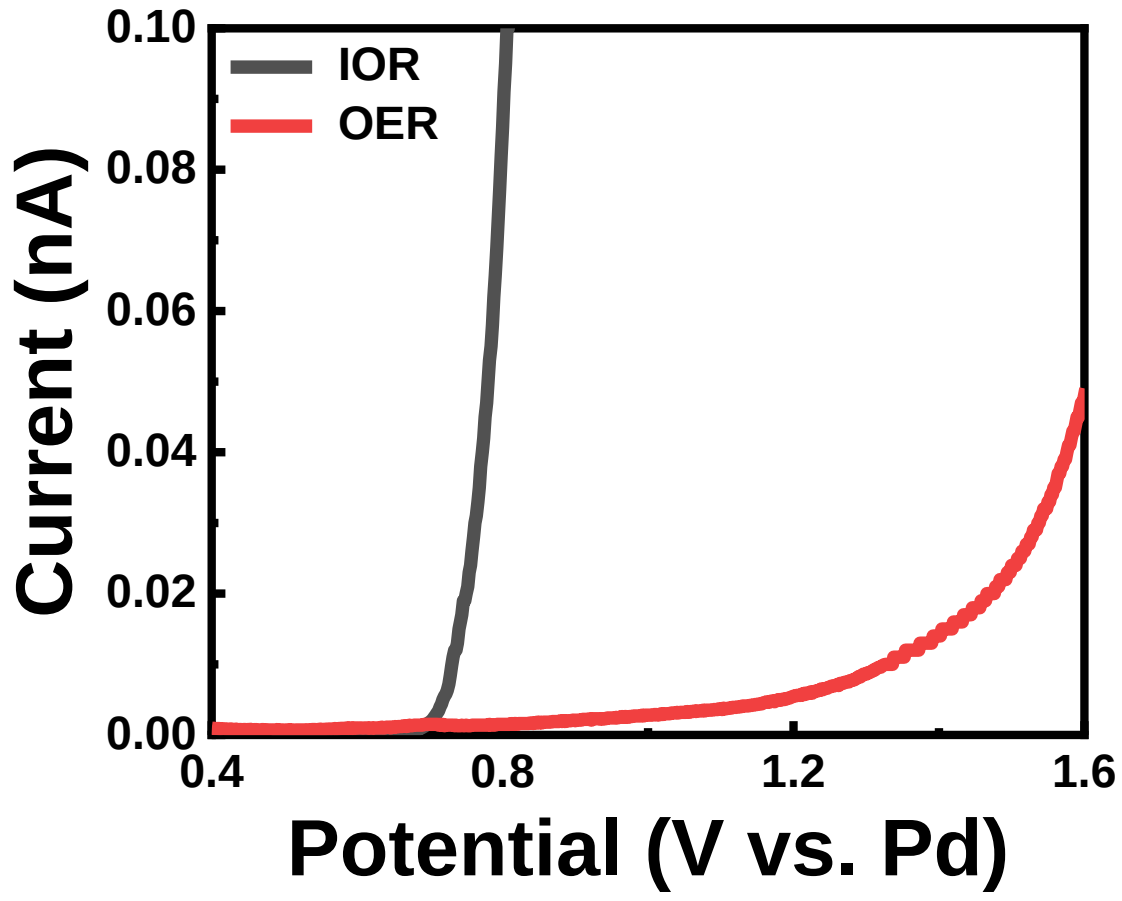

Figure S6. LSVs of CVD graphene for IOR (red) and OER (black). IOR shows a lower onset potential, indicating higher activity.

5. Twisted Bilayer Graphene IOR Electrocatalysis

Figure S7 shows the IOR Tafel slopes of MATBG, Bernal-stacked bilayer graphene, mechanically exfoliated monolayer graphene, and CVD-growth monolayer graphene. MATBG shows the smallest slope (55.0 mV dec^{-1}), confirming its superior catalytic performance toward IOR. By comparison, Bernal-stacked bilayer graphene exhibits a slope of 58.9 mV dec^{-1}, while mechanically exfoliated and CVD-grown monolayer graphene show very similar slopes of 59.2 mV dec^{-1} and 59.6 mV dec^{-1}, respectively.

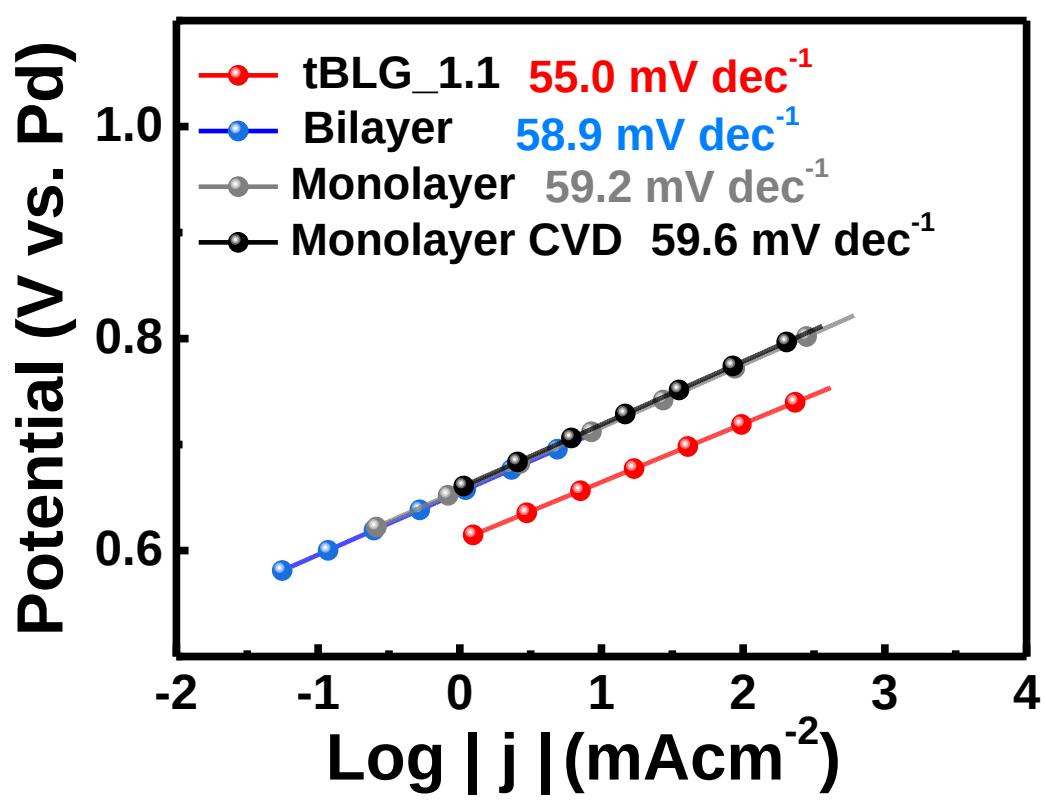

Figure S7. SECCM Tafel slope analysis comparing monolayer, bilayer, CVD-grown, and MATBG.

Figure S8(a) and (b) show localized SECCM measurements of LSV and Tafel slope at different regions of graphene, respectively. The basal plane exhibited higher current densities in LSV

measurements compared to edge regions. This is because the finite SECCM meniscus near the graphene boundary can introduce mixed contributions from the graphene edge and the adjacent inactive substrate region, therefore reducing the measured IOR current. By contrast, MATBG displayed even greater IOR activity relative to both the basal and edge regions of monolayer graphene. Representative LSVs show lower onset potentials and higher current densities for MATBG, while Tafel analysis indicates smaller slopes, reflecting improved charge-transfer kinetics.

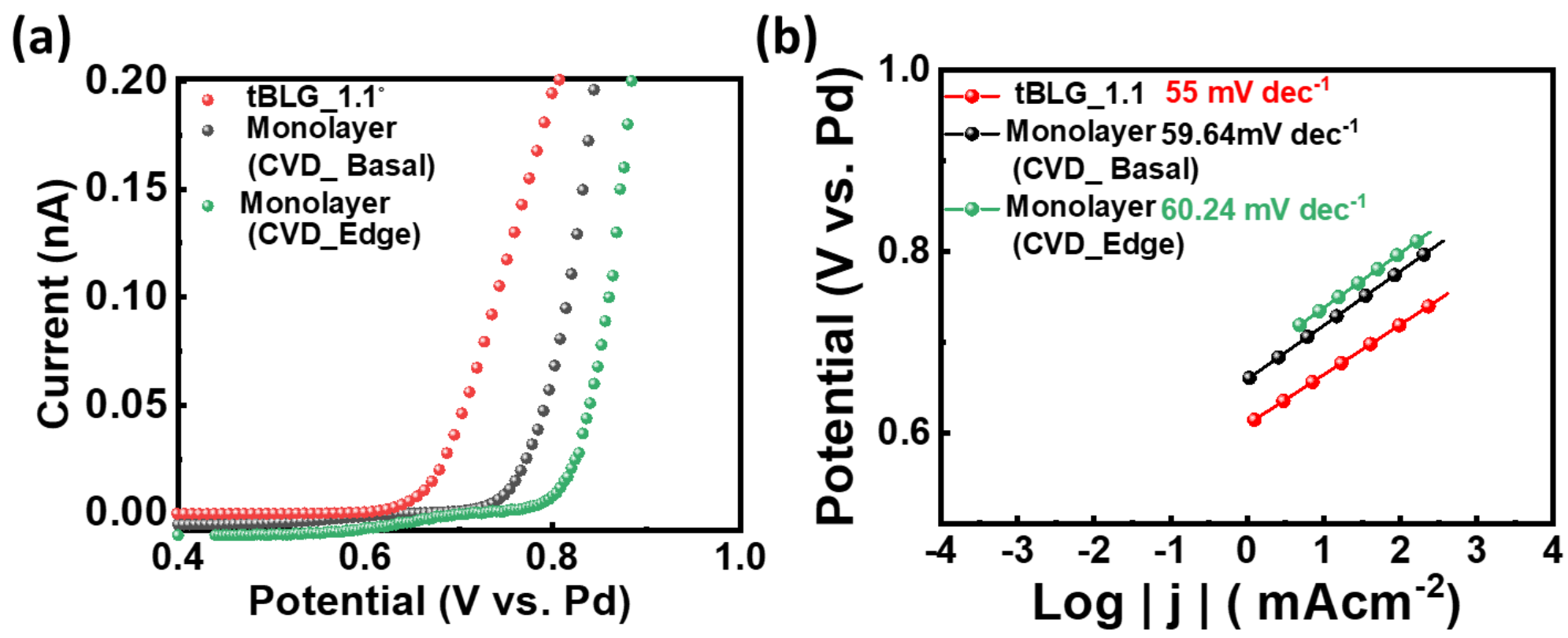

Figure S8. Local SECCM measurements of iodide oxidation reaction (IOR) on magic-angle twisted bilayer graphene (MATBG). (a) Representative linear sweep voltammograms (LSVs) comparing MATBG with basal and edge regions of monolayer graphene, showing lower onset potential and higher current densities for MATBG. (b) Corresponding Tafel plots indicating smaller Tafel slopes for MATBG, reflecting enhanced charge-transfer kinetics.

6. Device fabrication and characterization of the graphene field-effect transistor

Back-gated graphene field-effect transistors (FETs) were fabricated on n^+ Si substrates coated with a 90 nm SiO_2 layer, where the heavily doped silicon served as the global back gate and the SiO_2 layer functioned as the gate dielectric. A Ti/Pt layer was deposited to form the back-gate electrode. Monolayer graphene, transferred onto the substrate using the same wet-transfer procedure described previously, served as the conducting channel. Source and drain electrodes were defined using a shadow mask, and 7 nm Cr followed by 5 nm Au were deposited by thermal evaporation to complete the contacts, with a channel length of 100 µm and width of 200 µm. The fabricated graphene devices show consistent FET performance with slightly p-doped Dirac point as presented in Figure S9.

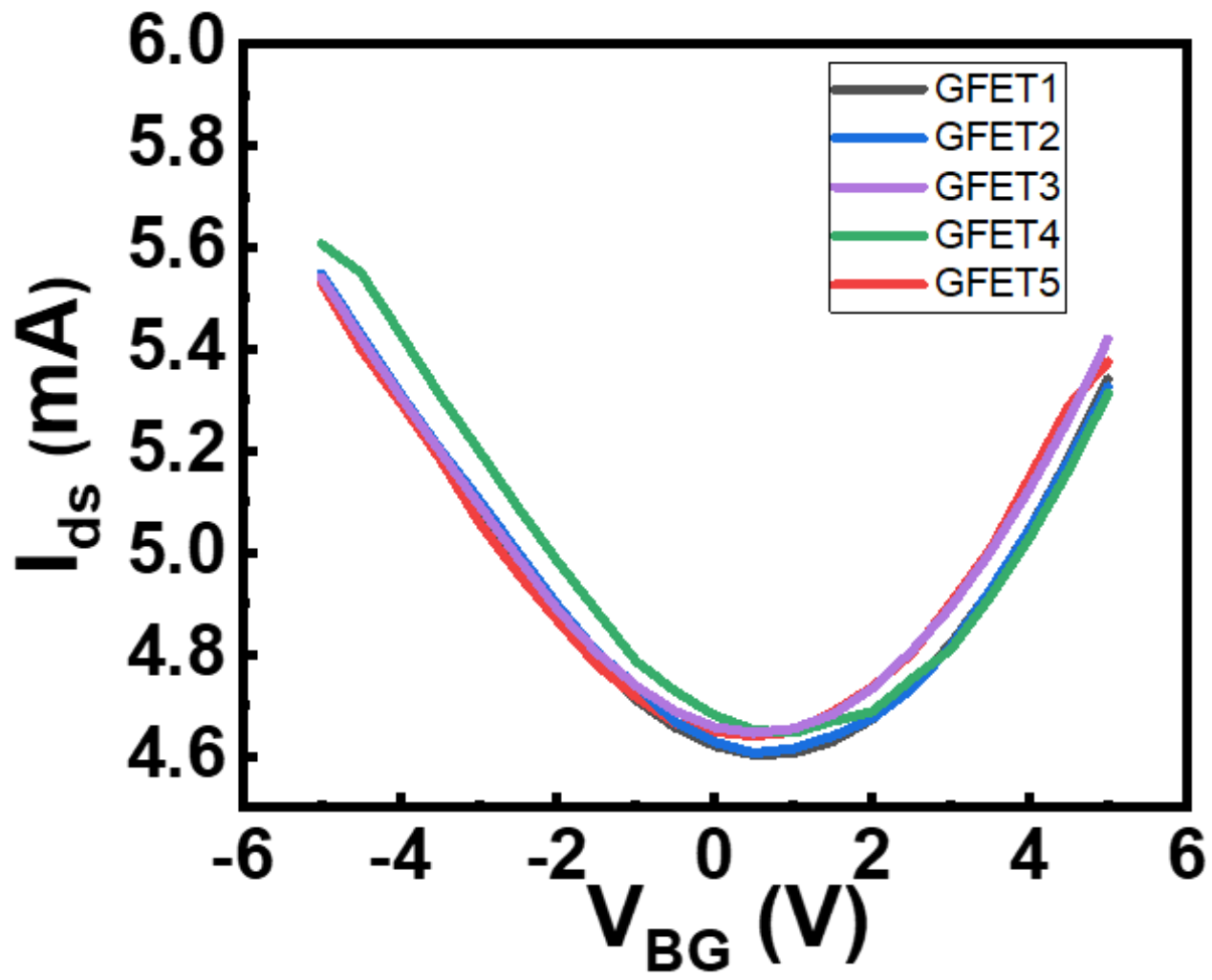

Figure S9. Transfer curves of 5 graphene FETs with the same architecture, fabricated and measured under conditions identical to those used for the device in the manuscript.

7. Modulation of graphene carrier concentration and Fermi level by electrostatic gating

The carrier concentration (n) of graphene was modulated through the control of the applied gate voltage of graphene FET, and is estimated by using the following equation：

$$n = \frac{C_g\left(V_g - V_{Dirac}\right)}{e}$$

C_g represents the gate capacitance per unit area and equals $\varepsilon_0\varepsilon_r/d$, where $\varepsilon_0 = 8.85 \times 10^{-12}$ Fm^{-1} is the vacuum permittivity, $\varepsilon_r = 3.9$ is the dielectric constant of SiO_2, d = 90 nm is the thickness of the dielectric layer. V_g is the applied voltage, V_{Dirac} is the gate voltage at the Dirac point and $e = 1.6 \times 10^{-19}$ C is the elementary charge. The corresponding shift in the Fermi level (E_F) of graphene can then be determined from the linear energy dispersion relation near the Dirac point:

$$E_F = \hbar V_F\sqrt{(\pi n)}$$

where ħ is the reduced Planck constant, $V_F \approx 1 \times 10^6$ m s^{-1} is the Fermi velocity of graphene, and n is the induced carrier concentration. Detailed numerical values are provided in the main text.

8. Device fabrication of TBG

High-quality monolayer, bilayer, and MATBG devices are fabricated using a standard polymer-based dry-transfer technique[1, 2], with a pre-patterned via hole in the top hexagonal

boron nitride (hBN) encapsulation serving as the viewport to enable direct electrochemical probing of iodide oxidation. Graphene and hBN flakes are first exfoliated onto a SiO_2/Si substrate, and suitable flakes are identified under an optical microscope. The fabrication process begins by patterning the top hBN flake with a square via window using electron beam lithography (EBL) and inductively coupled plasma (ICP) etching. This patterned hBN flake is picked up using a stamp composed of a dome-shaped PDMS covered with a PC film, and then used to sequentially pick up the van der Waals layers. For monolayer and bilayer graphene devices, the graphene flake is directly picked up by the top hBN. For MATBG devices, a tear-and-stack method is employed to sequentially pick up the two graphene layers with a controlled twist angle. The completed stack is then transferred onto a bottom hBN flake to finalize the dry assembly. Next, the one-dimensional edge contacts are patterned using EBL and ICP etching. Metal contacts are subsequently patterned by a second EBL step followed by plasma cleaning, and then deposited using Cr/PdAu. Once fabrication is completed, the chip is mounted on a leadless chip carrier and wire bonded.

9. Transmission Electron Microscopy Sample Preparation

The structural characteristics of the freestanding twisted bilayer graphene were investigated in a field-emission TEM (JEOL JEM-F200) operated at 80 keV and equipped with a Gatan Rio 16 camera. Before acquiring TEM images at room temperature, the twisted bilayer graphene was heated in the TEM at 500 K for 3 hours to remove carbon contamination and then naturally cooled down. Throughout many observed areas of customarily 1 μm^2 in the thus cleaned-up sample, neither ripples nor voids have been identified and no discernible electron beam damage has taken place during the observations. The thus-acquired TEM images were then subject to the Bragg filtering with the spatial frequency up to 0.469 $Å^{-1}$. The observed AA and AB stacking contrasts are robust to the underfocus imaging condition and also the Bragg filtering procedure. To further assess the lateral uniformity of the twisted bilayer graphene, selected-area electron diffraction (SAED) measurements were performed at five different positions across a 4 μm × 4 μm TBG area. As shown in the Figure S10, among these five positions, four exhibited a twist angle close to 1.1° (except for position 2), indicating that the magic-angle configuration is maintained over a laterally extended micrometer-scale region rather than being limited to a single isolated local point.

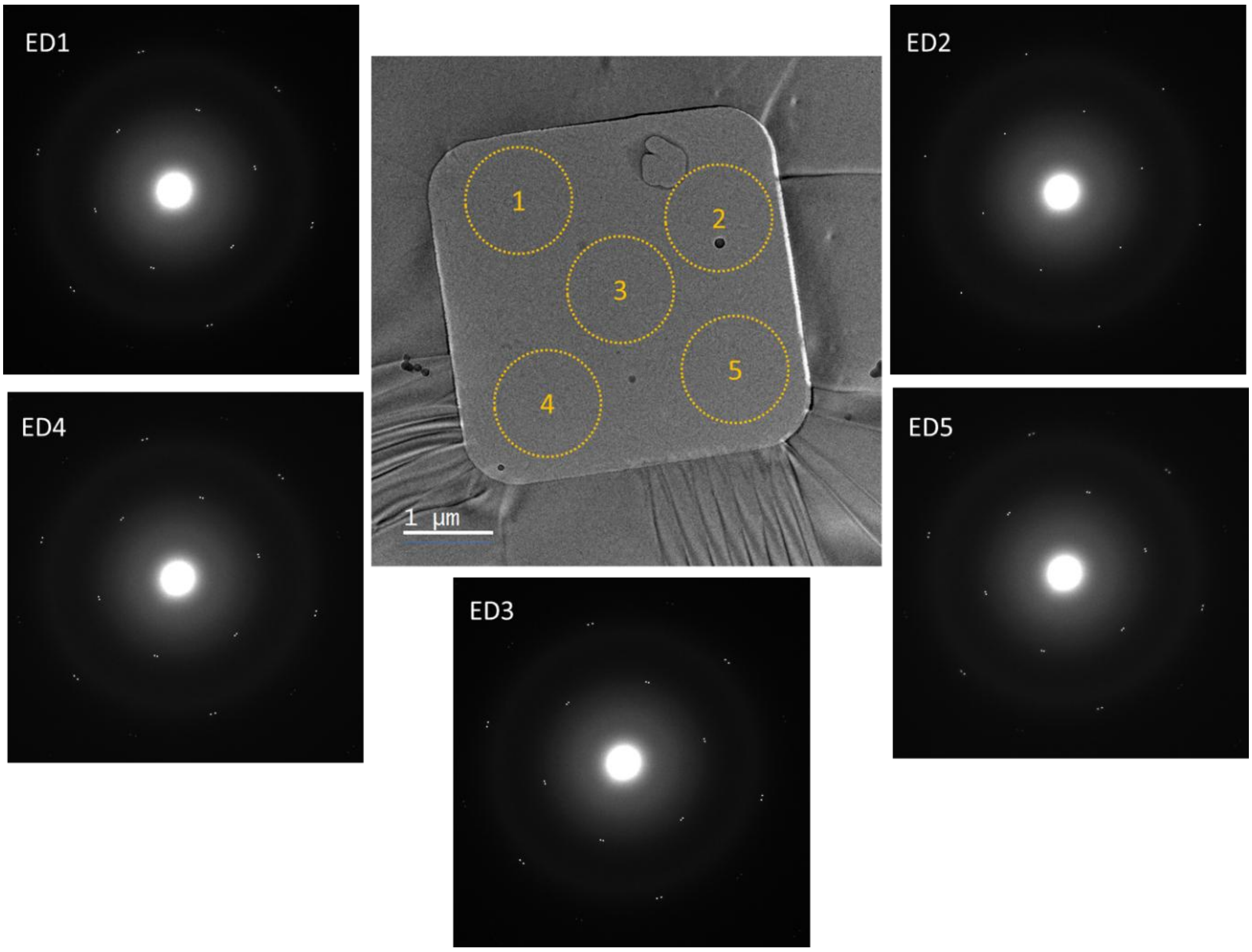

Figure S10. SAED patterns over 4 μm × 4 μm TBG sample.

10. Band structure calculation

Band structure of twisted bilayer graphene

The band structure of twisted bilayer graphene (tBLG) was computed using a plane-wave expansion of Bloch states around the corners of the Brillouin zone, $K_{\xi}^{(j)} = \xi K \cdot R_{(2\pi/3)j}(1,0)$, with $\mathcal{R}_{\phi}$ being the clockwise rotation operator of an angle $\phi, \xi = \pm 1$ is the valley index and $K = 4\pi/3a$ ($a \approx 2.46$ Å is graphene's lattice constant). This approach allows us to evaluate the explicit form of the matrix elements of the inter-layer coupling up to first order in momentum [3]. The full Hamiltonian in momentum representation takes the form

$$\mathcal{H} = \begin{pmatrix} H_g\left(\mathbf{k} - \frac{\Delta\mathbf{K}^{(0)}}{2}\right)\delta_{\mathbf{k}',\mathbf{k}} & \mathcal{T}_{\mathbf{k}',\mathbf{k}} \\ \mathcal{T}^{\dagger}_{\mathbf{k}',\mathbf{k}} & H_g\left(\mathbf{k} + \frac{\Delta\mathbf{K}^{(0)}}{2}\right) \end{pmatrix} \quad (1)$$

where the intra-layer Hamiltonian is the linearized Hamiltonian of graphene, $H_g(k) \equiv v\hbar(\xi k_x \sigma_x + k_y \sigma_y)$ ($v \approx 10^6$ m/s is the Fermi velocity of pristine graphene), while the inter-layer counterpart is

$$\mathcal{T}_{\mathbf{k}',\mathbf{k}} = \sum_{j=0}^{2}\left\{\left[\frac{\gamma_1}{3} + \frac{\hbar v_4}{3K}\,(\mathbf{k}'+\mathbf{k})\cdot\mathbf{K}_{\xi}^{(j)}\right]\begin{pmatrix} 1 & e^{\,i\xi\frac{2\pi}{3}j} \\ e^{-\,i\xi\frac{2\pi}{3}j} & 1 \end{pmatrix} + \,i\xi\,\frac{\hbar\,(v_3-v_4)}{3K}\,(\mathbf{k}'+\mathbf{k})\times \mathbf{K}_{\xi}^{(j)}\begin{pmatrix} 0 & e^{\,i\xi\frac{2\pi}{3}j} \\ -\,e^{-\,i\xi\frac{2\pi}{3}j} & 0 \end{pmatrix}\right\}\,\delta_{\mathbf{k}',\,\mathbf{k}+\Delta\mathbf{K}_{\xi}^{(0)}-\Delta\mathbf{K}_{\xi}^{(j)}} \tag{2}$$

Above, we introduce the dimer bond $\gamma1 = 0.39\,eV, and\ (v3, v4) = (0.10, 0.044)v$, as well as the corners of the mini-Brillouin zone (mBZ), $\Delta K_{\xi}^{(j)} \equiv \theta z \times K_{\xi}^{(j)}$. The Hamiltonian in Eq. (1) can be diagonalized in the basis of Bloch states of the top and bottom layers, centered around the reciprocal superlattice vectors $\Delta G = n\Delta G_{\xi}^{(1)} + m\Delta G_{\xi}^{(2)}$, where$\Delta G_{\xi}^{(1,2)} \equiv \Delta K_{\xi}^{(0)} - \Delta K_{\xi}^{(1,2)}$. The basis, in principle infinite, is truncated to include all reciprocal superlattice vectors up to a high enough cutoff value $|\Delta G| \le k_c$ that ensures convergence for all points in the first mBZ.

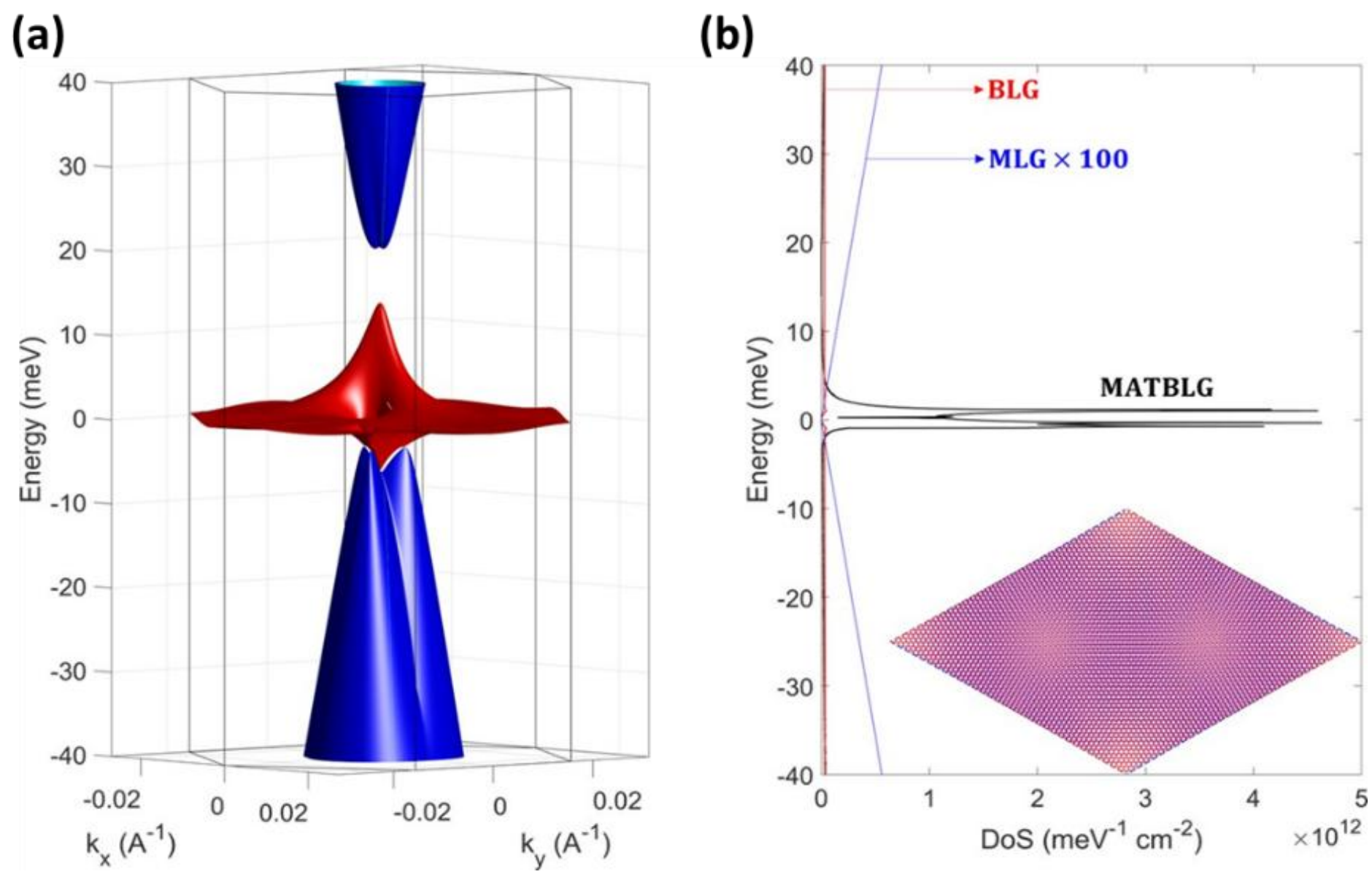

Figure S11. (a)Band structure of twisted bilayer graphene and (b) density of states (DoS). For comparison, we also plotted the DoS of bilayer graphene (BLG) in red and monolayer graphene (MLG) in blue, the latter magnified 100 times.

To obtain the density of states (DoS), $\rho(E)$, we first calculate the eigenvalues of the Hamiltonian in Eq. (1), $\epsilon_{\xi,k}^{\beta}$, where β labels the band index, and compute numerically the integral

$$\rho(E) = 2\sum_{\xi,\beta}\int \frac{dk}{(2\pi)^2}\,\delta\left(E - \varepsilon_{\xi,k}^{\beta}\right) \tag{3}$$

In Fig. S11(a), we plot the dispersion of twisted bilayer graphene for $\theta = 1.05°$, often referred to as magic-angle twisted bilayer graphene (MATBLG). The two bands closest to the charge neutrality point, colored in red, concentrate almost all their eigenvalues at an energy window of about ~ 3 meV. The flatness of the bands is best illustrated in panel (b) of the same figure, where we plot the DoS for twisted bilayer graphene. To put in perspective the band flattening in MATBLG, we also compute the density of states of suspended monolayer graphene, magnified by 100 times, and bilayer graphene, which also feature and enhancement of the density of states due to the van Hove singularity.

Projected density of states

Band flattening is always linked to some form of localization in real space. To gain insights on this feature of the electronic spectrum, here we study the wave function composition of the flat bands. We first define the moiré unit cell, a parallelogram spanned by the two moiré lattice vectors $R_m^{1,2} = R_m\left(\frac{\sqrt{3}}{2}, \pm\frac{1}{2}\right)$, where $R_m = a/\theta$ is the moiré lattice constant. Then the spatial distribution of charges associated to each point in the band structure, $\epsilon_{\xi,k}^{\beta}$, can be expressed in terms of the components of its associated eigenvector [4]

$$n_{\xi,k}^{\beta}(r) = \sum_{\lambda} \left|\sum_{\Delta G} c_{\lambda\xi k}^{\beta}(\Delta G) \cdot e^{-i\Delta G\cdot r}\right|^2 \tag{4}$$

where λ labels the layer and sublattice. To visually illustrate the expression above, we show in Fig. S12 the charge distribution associated to three eigenvalues of the first conduction band, located at $\gamma = (0,0), \kappa = (0, \theta K)$ and $\mu = \left(\theta K \frac{\sqrt{3}}{2}, 0\right)$. We note that points around which the band structure is flatter, like κ *and* μ, charges tend to locate around the corners of the moire unit cell, where tBLG feature locally an AA-stacking configuration. Conversely, for points in the mBZ where the first conduction flat band is more dispersive, like the γ-point, the charges are relatively more delocalized and exhibit non-zero amplitude at most of the moire unit cell.

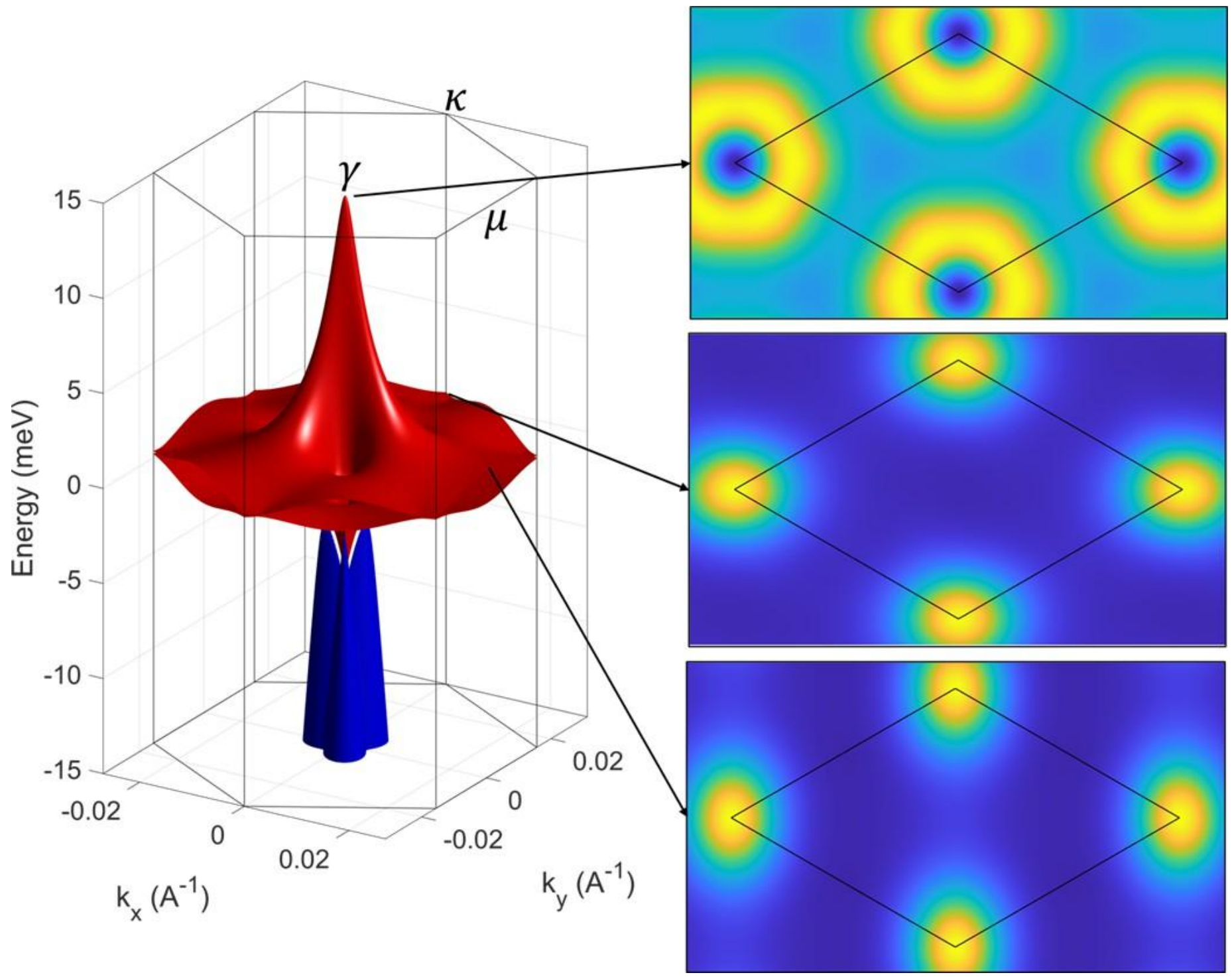

Figure S12. (Left) Band structure of magic-angle twisted bilayer graphene in a 30 meV energy window around the charge neutrality point. (Right) spatial distribution of charges around at the γ (top), κ (middle) and μ (bottom) points of the first conduction flat band.

Finally, to quantify the effect of localization on the density of states, we define three circular regions, Ω_{AA}, Ω_{AB} *and* Ω_{BA} within the moire unit cell of radius $R_m/8$ centered at $r = 0, r = R_m/\sqrt{3}(1,0)$, and $r = 2R_m/\sqrt{3}(1,0)$, points that correspond to a locally AA, AB and BA stacking, respectively (see Fig. S11). We then define a weight to each point in the band structure based on its projection onto these three patches,

$$w^{\beta}_{\xi,k,X} = \int_{\Omega_X} n^{\beta}_{\xi,k}(r)dr\,,\ X = AA, AB, BA \tag{5}$$

and re-evaluate the density of states in Eq. (6) including these weights,

$$\rho_X(E) = 2\sum_{\xi,\beta}\int d\,k\,/(2\pi)^2 \cdot w^{\beta}_{\xi,k,X} \cdot \delta\left(E - \varepsilon^{\beta}_{\xi,k}\right),\ X = AA, AB, BA \tag{6}$$

In Fig. S13, we present the band structure of tBLG with opacity proportional to its projection to the Ω_{AA}, Ω_{AB} and Ω_{BA}, as well as the projected DoS for these three circular regions. As mentioned earlier, the density of states of the flat bands, colored in red, is mostly localized around the regions in the moiré unit cell featuring locally an AA stacking configuration locally.

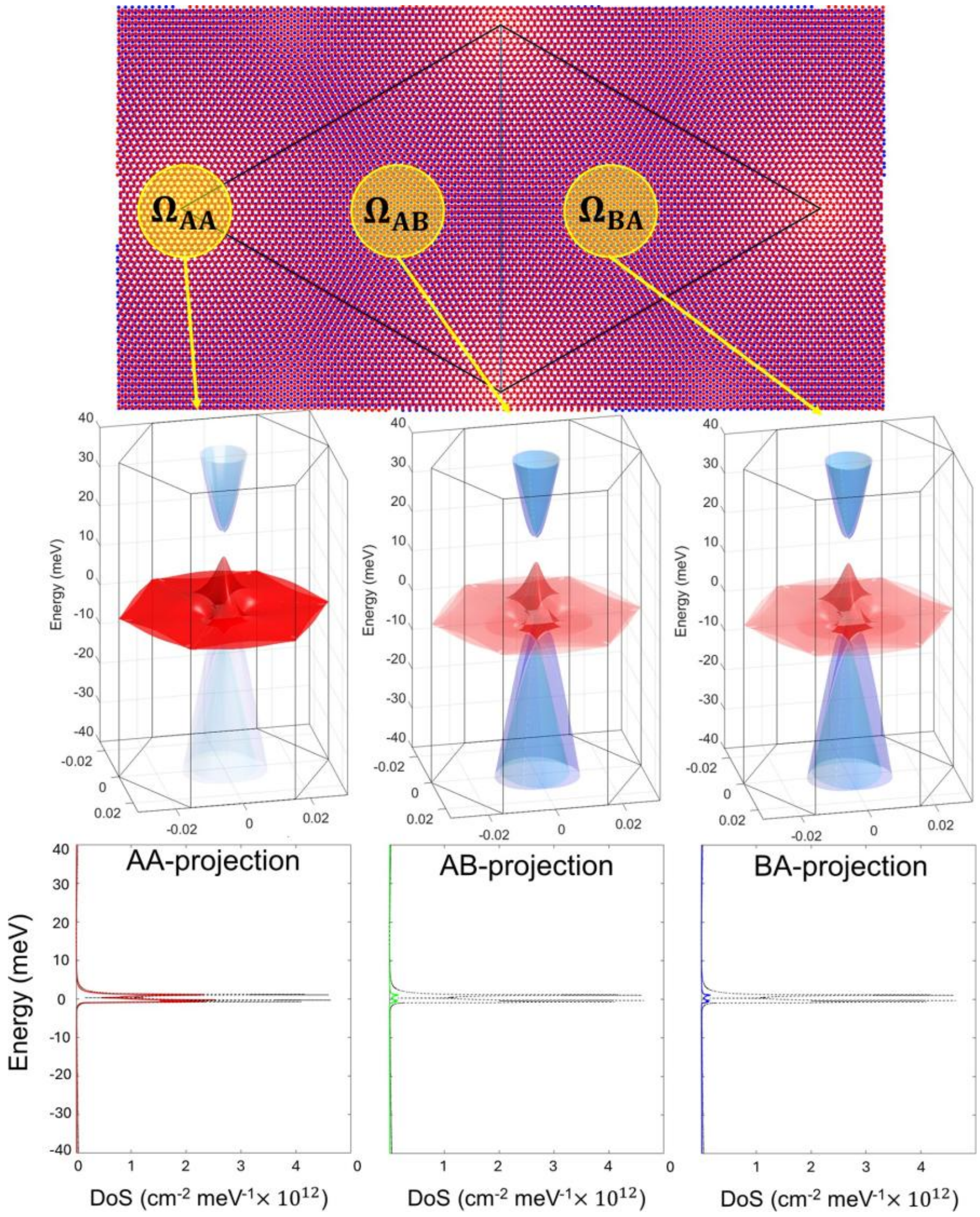

Figure S13. In the upper panel, we show a rectangular portion of magic angle twisted bilayer graphene, enclosing the moiré unit cell and highlighting the regions in the spectrum where the stacking is locally AA, AB and BA. In the band structure, shown in the middle panel, the opacity of each point is proportional to its projections to these circular regions. In the lower panel, we present the DoS projected to these regions.

11. Statistic of replicated SECCM measurements on graphene

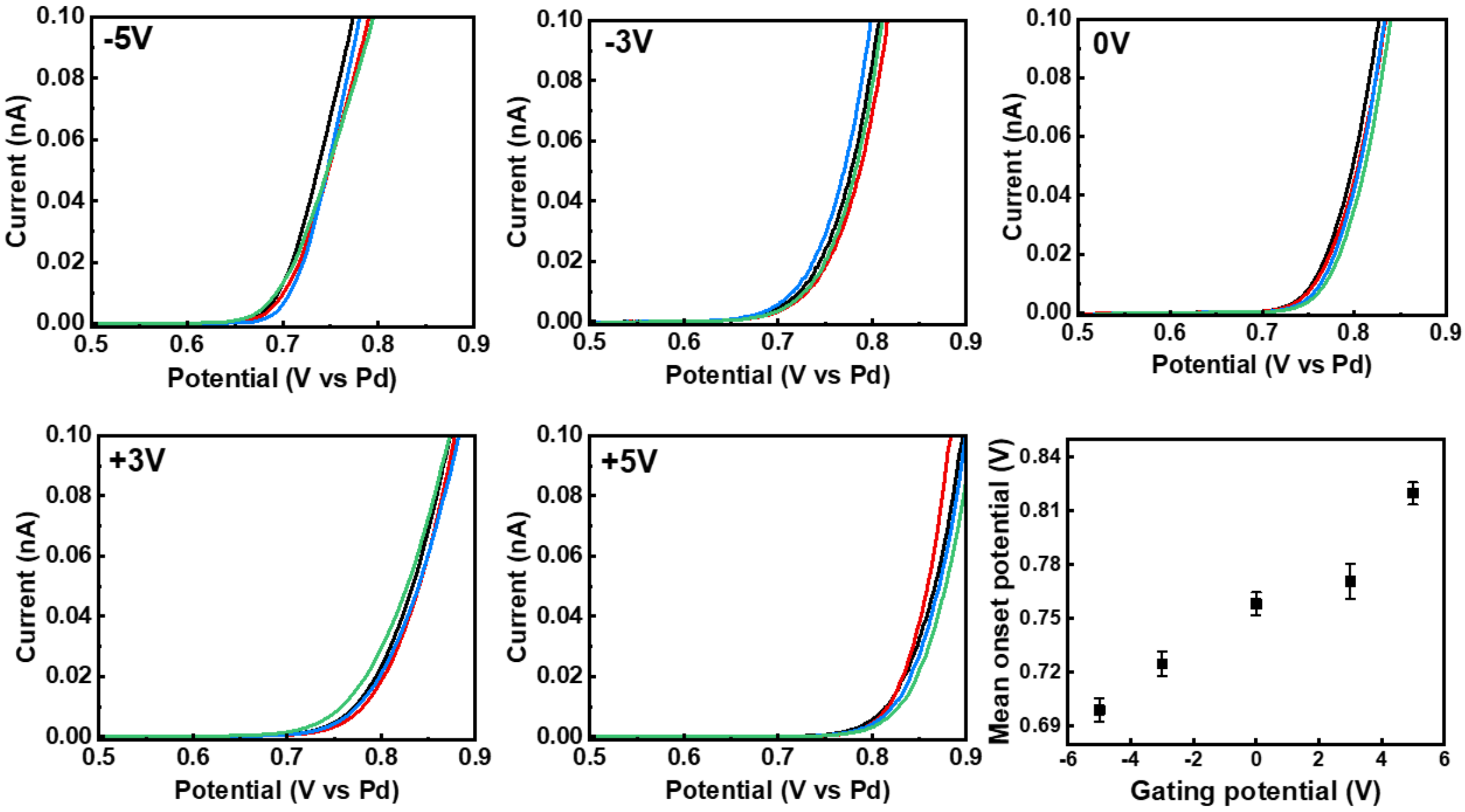

Figure S14 Replicated measurement of graphene IOR corresponds to Figure 2c in manuscript.

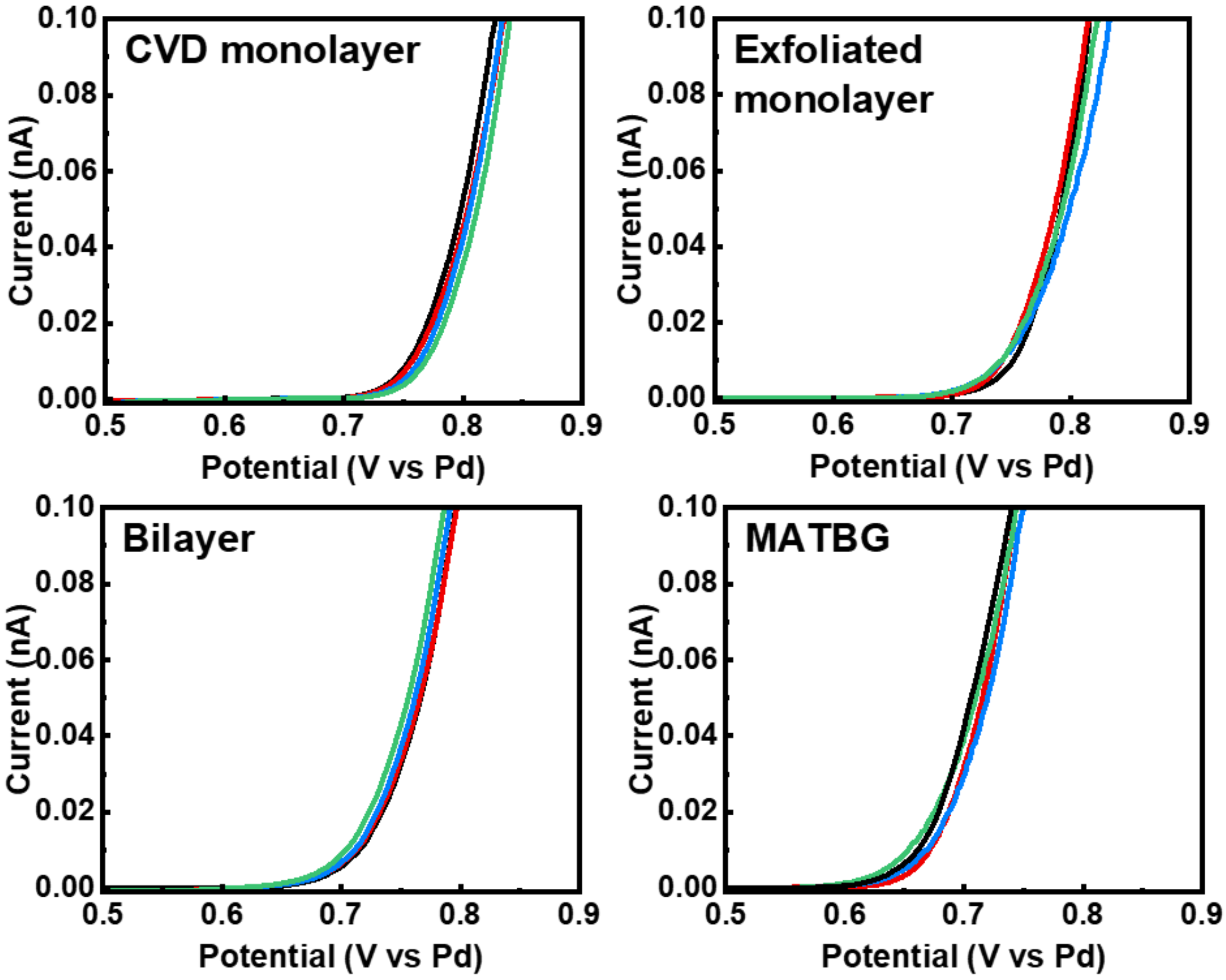

Figure S15. Replicated SECCM measurement of IOR corresponds to Figure 4c in manuscript.

Table S1. Statistic data of all SECCM IOR samples

Graphene sample	Mean onset potential (V)	SD (V)	Mean Tafel slope (mV/dec)	SD (mV/dec)
+5V	0.82	0.009	65.57	3.43
+3V	0.77	0.012	65.37	4.21
-3V	0.72	0.015	61.42	2.70
-5V	0.69	0.011	54.13	3.71
CVD monolayer (0V)	0.75	0.010	59.25	2.50
MATBG	0.66	0.006	54.66	2.91
Bilayer	0.71	0.008	57.75	2.65
Exfoliated monolayer	0.74	0.006	58.59	2.47

Statistical analysis was performed to evaluate the reproducibility and distinguishability of the local SECCM responses. For multiple-group comparisons, one-way ANOVA followed by Tukey's HSD post-hoc test was used. A p-value less than 0.05 was considered statistically significant. Significance levels are denoted as $^{*}p < 0.05$, $^{**}p < 0.01$, and $^{***}p < 0.001$; n.s. indicates no statistically significant difference. One-way ANOVA followed by Tukey's HSD post-hoc test shows that the onset potential varies significantly with gate voltage at the local measurement level, $F(4,15) = 73.02$, $p < 0.001$. The negative gate biases consistently shift the onset potential to lower values compared with the positive gate biases, supporting the conclusion that hole-doped graphene promotes IOR under the present SECCM configuration. In addition, one-way ANOVA for the graphene-structure comparison shows that the local onset potentials are statistically distinguishable among the investigated graphene structures, $F(3,12) = 110.75$, $p < 0.001$. Tukey's post-hoc analysis confirms that MATBG exhibits a significantly lower local onset potential than Bernal-stacked bilayer graphene and monolayer graphene. The analyzed statistical results of gating effect and structure effect are shown in the **Figure S16** (a) and (b), respectively.

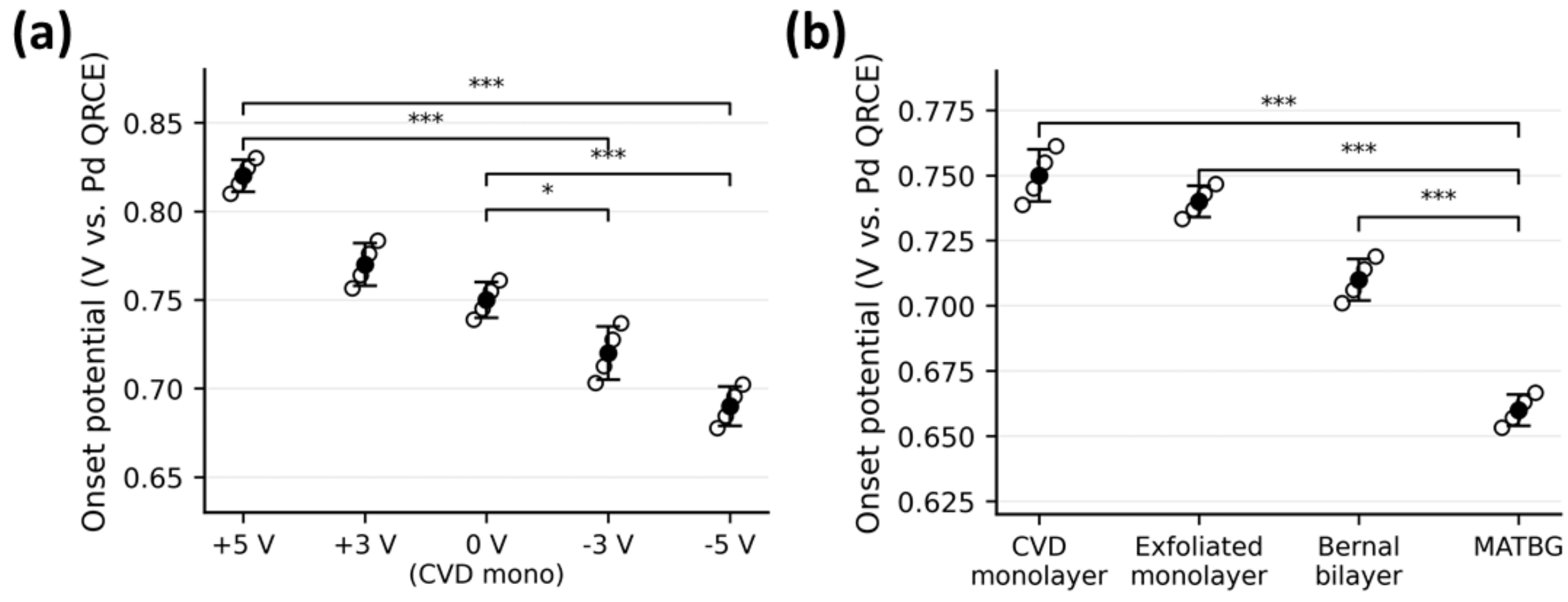

Figure S16. Statistical analysis of (a) gating dependence and (b) structure dependence of graphene IOR onset potentials.

12. Twist-angle homogeneity assessment

To further assess the twist-angle homogeneity achievable with our fabrication process, we performed dual-gate transport measurements on MATBG devices fabricated using the same heterostructure assembly setup, equipment, and processing procedures as those used for the SECCM devices. This approach provides a sensitive benchmark for evaluating the twist-angle uniformity achievable with our fabrication process. Since there is currently no direct method to quantify the twist-angle uniformity in the original SECCM devices, measurements on transport devices fabricated under nominally identical conditions provide a useful complementary assessment of homogeneity for this device platform.

As shown in the **Figure S17**, longitudinal resistance was measured from three spatially distinct regions of the MATBG transport device, corresponding to the top (1-2), middle (2-3), and bottom (3-4) regions. All three regions exhibit well-defined resistance maxima at the charge neutrality point and at the satellite filling densities. The satellite peaks are centered at approximately $n = \pm 3.237 \times 10^{12}$ cm^{-2}, corresponding to complete filling of four electrons per moiré unit cell and confirming the presence of a well-defined moiré superlattice. The local twist angle θ was extracted from the satellite density n_s, using

$$n_s = \frac{8}{\sqrt{3}a^2}\theta^2$$

, where a is the graphene lattice constant.[5, 6] The blue shaded regions in **Figure S17** indicate the range of ns corresponding to a twist-angle variation of ±0.05°. The satellite features measured from all three regions remain within this narrow range, yielding a consistent twist angle of θ = 1.18° ± 0.05°. Since the observed spread of the satellite peaks is smaller than the range defined by ±0.05°, these measurements indicate that the twist-angle variation is below 0.05° over micrometer length scales. This result supports good twist-angle homogeneity and the reproducibility of the fabrication procedure used for the SECCM devices.

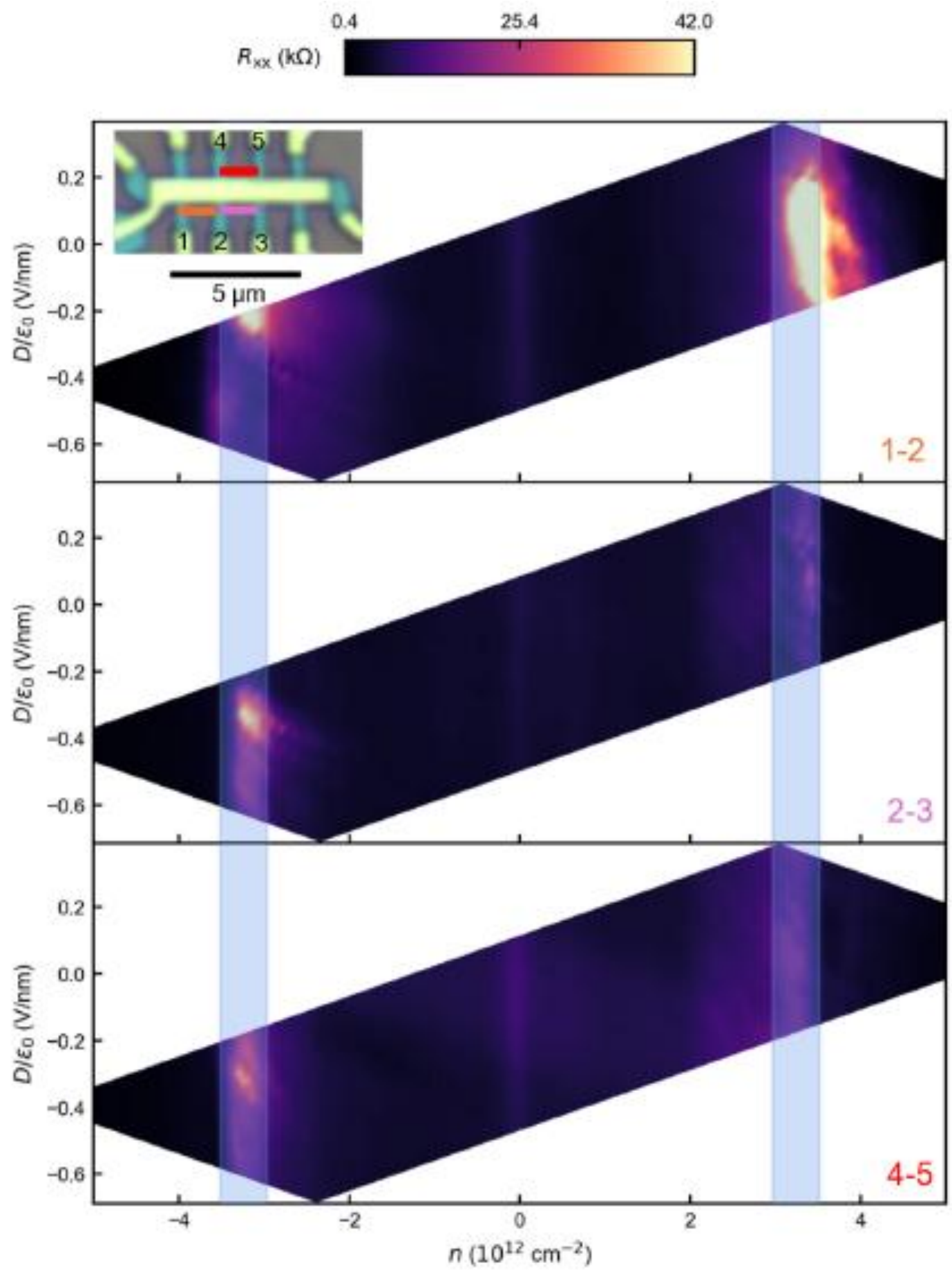

Figure S17. Longitudinal resistance as a function of displacement field and carrier density. Measurements were performed in a dilution refrigerator with a base temperature of T = 26 mK using different contact pairs corresponding to the top (1–2), middle (2–3), and bottom (4–5) regions of the device. Inset: optical micrograph of the device with numbered contacts. Colored traces denote the corresponding contact configurations.

13. Electrostatic gating effect on Ru(NH3)6^{3+} reduction

To validate the gate-dependent charge-transfer behavior, Ru(NH3)6^{3+} reduction was remeasured using the same nanopipette pre-screening protocol applied to the IOR experiments. The plateau currents remain comparable under different gate biases, indicating that the mass-transport-limited current is primarily determined by the meniscus geometry and is not significantly affected by the applied gate voltage. In contrast, the onset potential shifts systematically with gate bias. In particular, applying a positive gate bias lowers the onset potential for Ru(NH3)6^{3+} reduction, consistent with enhanced electron donation from electron-doped graphene to the redox species. This trend is opposite to that observed for IOR, where negative gate bias promotes electron extraction from iodide and enhances iodide oxidation. This behavior is consistent with enhanced electron donation from electron-doped graphene to $Ru(NH_3)_6^{3+}$ and supports the role of electrostatic gating in modulating interfacial charge-transfer energetics.

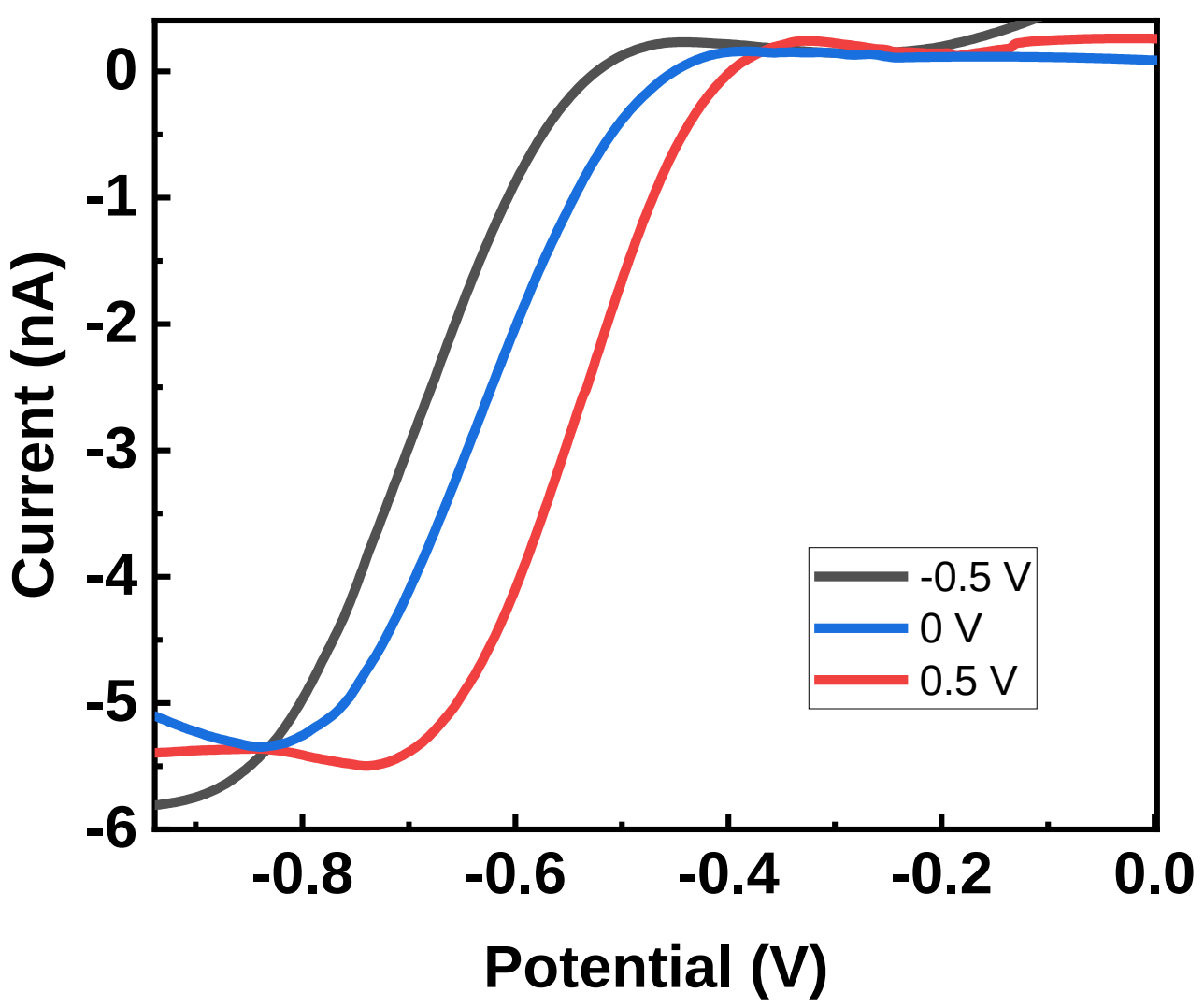

Figure S18. Gate-dependent Ru(NH3)6^{3+} reduction.

14. References

(1) Cao, Y.; Luo, J.; Fatemi, V.; Fang, S.; Sanchez-Yamagishi, J.; Watanabe, K.; Taniguchi, T.; Kaxiras, E.; Jarillo-Herrero, P. Superlattice-induced insulating states and valley-protected orbits in twisted bilayer graphene. *Physical review letters* **2016**, *117* (11), 116804.

(2) Kim, K.; Yankowitz, M.; Fallahazad, B.; Kang, S.; Movva, H. C.; Huang, S.; Larentis, S.; Corbet, C. M.; Taniguchi, T.; Watanabe, K. van der Waals heterostructures with high accuracy rotational alignment. *Nano letters* **2016**, *16* (3), 1989-1995.

(3) Garcia-Ruiz, A.; Deng, H.-Y.; Enaldiev, V. V.; Fal'ko, V. I. Full Slonczewski-Weiss-McClure

parametrization of few-layer twistronic graphene. *Physical Review B* **2021**, *104* (8), 085402.
(4) Garcia-Ruiz, A.; Liu, M.-H. Twisted Bilayer MoS2 under Electric Fields: A System with Tunable Symmetry. *Nano letters* **2024**, *24* (51), 16317-16324.
(5) Cao, Y.; Fatemi, V.; Fang, S.; Watanabe, K.; Taniguchi, T.; Kaxiras, E.; Jarillo-Herrero, P. Unconventional superconductivity in magic-angle graphene superlattices. *Nature* **2018**, *556* (7699), 43-50.
(6) Lu, X.; Stepanov, P.; Yang, W.; Xie, M.; Aamir, M. A.; Das, I.; Urgell, C.; Watanabe, K.; Taniguchi, T.; Zhang, G. Superconductors, orbital magnets and correlated states in magic-angle bilayer graphene. *Nature* **2019**, *574* (7780), 653-657.
